# Structure-Based In Silico Screening of Marine Phlorotannins for Potential Walrus Calicivirus Inhibitor

**DOI:** 10.3390/ijms242115774

**Published:** 2023-10-30

**Authors:** Nalae Kang, Eun-A Kim, Seong-Yeong Heo, Soo-Jin Heo

**Affiliations:** Jeju Bio Research Center, Korea Institute of Ocean Science and Technology (KIOST), Jeju 63349, Republic of Korea; nalae1207@kiost.ac.kr (N.K.); euna0718@kiost.ac.kr (E.-A.K.); syheo@kiost.ac.kr (S.-Y.H.)

**Keywords:** in silico, phlorotannin, walrus calicivirus

## Abstract

A new calicivirus isolated from a walrus was reported in 2004. Since unknown marine mammalian zoonotic viruses could pose great risks to human health, this study aimed to develop therapeutic countermeasures to quell any potential outbreak of a pandemic caused by this virus. We first generated a 3D model of the walrus calicivirus capsid protein and identified compounds from marine natural products, especially phlorotannins, as potential walrus calicivirus inhibitors. A 3D model of the target protein was generated using homology modeling based on two publicly available template sequences. The sequence of the capsid protein exhibited 31.3% identity and 42.7% similarity with the reference templates. The accuracy and reliability of the predicted residues were validated via Ramachandran plotting. Molecular docking simulations were performed between the capsid protein 3D model and 17 phlorotannins. Among them, five phlorotannins demonstrated markedly stable docking profiles; in particular, 2,7-phloroglucinol-6,6-bieckol showed favorable structural integrity and stability during molecular dynamics simulations. The results indicate that the phlorotannins are promising walrus calicivirus inhibitors. Overall, the study findings showcase the rapid turnaround of in silico-based drug discovery approaches, providing useful insights for developing potential therapies against novel pathogenic viruses, especially when the 3D structures of the viruses remain experimentally unknown.

## 1. Introduction

The coronavirus disease 2019 (COVID-19) pandemic highlighted the significance of structure prediction and interaction models on the development of effective drugs [1]. The World Health Organization (WHO) has listed several priority diseases for which there are either no or insufficient medical countermeasures and therefore are at risk of causing future epidemics. The WHO has also issued warnings for “Disease X”, a disease caused by an unknown pathogen that is not yet known to cause any illness in humans [2,3,4]. Following the COVID-19 era, international responses to any unknown viral infections that may occur in the future are being outlined in detail, as this is becoming an increasingly relevant topic of discussion [2]. Accurate structure prediction of proteins present in the pathogens can help accelerate drug discovery so that relevant authorities may be better prepared for future epidemics [2].

The marine environment is a reservoir of approximately 10^30^ viruses that infect a wide range of organisms, from shrimp to whales, and lead to disease and mortality in these organisms [5,6]. Recent studies have revealed that marine virus ecosystems—including biogeochemical cycles, food networks, and the metabolic balance of the ocean—are affected by various factors such as climate change, sea ice melting, and marine acidification [7]. This indicates that several viruses may emerge from the marine ecosystem as new human viral infectious agents, which necessitates increased research on effective countermeasures, especially for viruses that are in close contact with humans.

Marine viruses belong to various viral families—such as Coronaviridae, Picornaviridae, Paramyxoviridae, Rhabdoviridae, and Caliciviridae—that cause several human diseases, such as severe acute respiratory syndrome (SARS) and coronavirus disease 2019, middle east respiratory syndrome, laryngopharyngitis, mumps, measles, respiratory tract disease, flu-like symptoms, and gastroenteritis. According to a previous study, unknown marine mammalian zoonotic viruses could pose great risks to human health [8]. Several virus families that may threaten human health have been isolated from marine mammals, such as *Delphinapterus* sp. (beluga whale; Coronaviridae, [9]), *Pusa sibirica* and *Phoca vitulina vitulina* (seal; Paramyxoviridae, [10]), and *Phocoena phocoena* (porpoise; Rhabdoviridae, [11]). Caliciviridae is a family of small, spherical viruses that infect vertebrates and have been found in humans as well as several companion and livestock animals, such as cats, cattle, pigs, and chickens. Feline calicivirus (FCV) causes respiratory illness and stomatitis in cats. Further, Caliciviridae are a class of human noroviruses that are a leading cause of infectious acute gastroenteritis through seafood intake. FCV is used as a surrogate model for noroviruses. Moreover, noroviruses have been shown to cross species, and a previous study reported the case of a San Miguel sea lion virus infecting a human [12]. Therefore, studies on marine caliciviruses are required to minimize their risks.

In 2004, a study reported the isolation of a new calicivirus from the walrus (*Odobenus* sp.), and its amino acid sequences were analyzed [13]. The first step to facilitate the in silico prediction of potential inhibitors against the emerging caliciviruses from the marine environment is the generation of 3D structures of the virus proteins based on their amino acid sequence information. Caliciviruses are non-enveloped icosahedral viruses. The capsid of FCV is assembled from 90 dimers of the major capsid protein (VP1), consisting of three domains: the N-terminal arm (NTA), the shell domain (S domain), and the protruding domain (P domain). Among them, the P domain forms the characteristic arch-shaped spikes on the virion surface, and plays a role in attachment to the host’s receptor, feline junctional adhesion molecule A (fJAM-A). FCV binds to fJAM-A through the outer face of the P domain of VP1; thereafter, the portal assembly comprising 12 copies of the VP2 protein is altered, leading to the opening of a pore in the capsid shell, and virions are finally uncoated. Therefore, the inhibition of receptor engagement of the P2 domain of VP1 can be used to inhibit FCV entry. Consequently, one emerging strategy for the inhibition of viral attachment is developing inhibitors that can engage single or several of the binding sites [14,15].

Natural products, broadly defined as chemicals produced by living organisms, have evolved over millions of years to acquire unique chemical diversity, resulting in a wide range of biological activities and drug-like properties [16]. They have been used as active components in many traditional medicine systems and have become one of the most important resources for the development of new lead compounds in contemporary drug discovery [17]. Between 1981 and 2014, more than 50% of drugs developed were based on natural products [16].

Phlorotannins are a group of polyphenols found in brown seaweeds that exhibit a range of biological activities, such as antioxidant, anti-inflammatory, and anticancer activities, depending on the position and number of their hydroxyl groups and aromatic rings [18,19]. Many studies have indicated that phlorotannins also exert antiviral effects [20,21,22]; dieckol and phlorofucofuroeckol-A have been confirmed to exert antiviral effects against Caliciviridae in vitro [21]. Therefore, screening phlorotannins for antiviral activity is a particularly interesting topic, considering their biological relevance as well as their intrinsic chemical and structural diversity.

Therefore, the present study aimed to generate a 3D structure of the walrus calicivirus capsid protein and screen several phlorotannin candidates occurring in marine natural products for identifying potential walrus calicivirus inhibitors.

## 2. Results

### 2.1. 3D Structure of the Walrus Calicivirus Capsid Protein Homology Model

Many recent studies have elucidated the 3D structures or the relevant homology models of several viruses, including SARS-CoV-2 [23,24,25,26]. This is part of the ongoing effort to expedite drug discovery when drug candidates can be identified based on such 3D-modeling approaches already available in the existing literature. To identify candidates against walrus calicivirus, the capsid protein structure was modeled using a homology modeling program. The quality of the homology model was dependent on sequence identity and similarity between the target and templates, followed by an observation that protein structures are better conserved than amino acid sequences [27]. It was seen that if the target exhibits 30–50% identity to templates, the model typically shows the identity and/or similarity in the general structure of the protein, with considerable differences in the loops of the protein. Thus, using this approach and setting our threshold for sequence identity between 30 and 50%, templates for homology modeling were selected [28]. Two PDB templates (PDB IDs: 2GH8 and 6GSI) were selected as references, and the target sequence exhibited 31.3% identity and 42.7% similarity to these templates (Figure S1). A strictly conserved Gly414 at the P1/P2 junction and P2 subdomain containing six beta-strands were maintained in the capsid homology model [29]. The variations in sequence between the target and two template proteins were predominantly observed in the loops, including a poly(A)-tail at NTA. In case of comparing each species, the walrus calicivirus capsid protein showed 61.4% identity and 75.3% similarity to sea lion calicivirus capsid protein (PDB ID: 2GH8), and 48.4% identity and 64.1% similarity to FCV capsid protein (PDB ID: 6GSI). Additionally, the walrus calicivirus capsid protein showed 47.7% identity and 63.3% similarity to FCV capsid protein (PDB ID: 3M8L) (Figure S1b).

In total, 20 models of the walrus calicivirus capsid protein were generated based on the aligned target sequence using the template protein sequences. Probability density function (PDF) and discrete optimized protein energy (DOPE) scores were used to select the most reliable model. These energy measurements provide a helpful readout for comparing the different models, with low energy values corresponding to an increased likelihood of the predicted tertiary structure of the model representing the in vivo conformation with a large degree of confidence [30]. The lowest PDF total energy and physical energy values obtained from the generated 3D homology model were 20,760 and 1699.07 kcal/mol, respectively (Figure S2 and Table S1). This model was used for all subsequent analyses.

**Figure 1:**
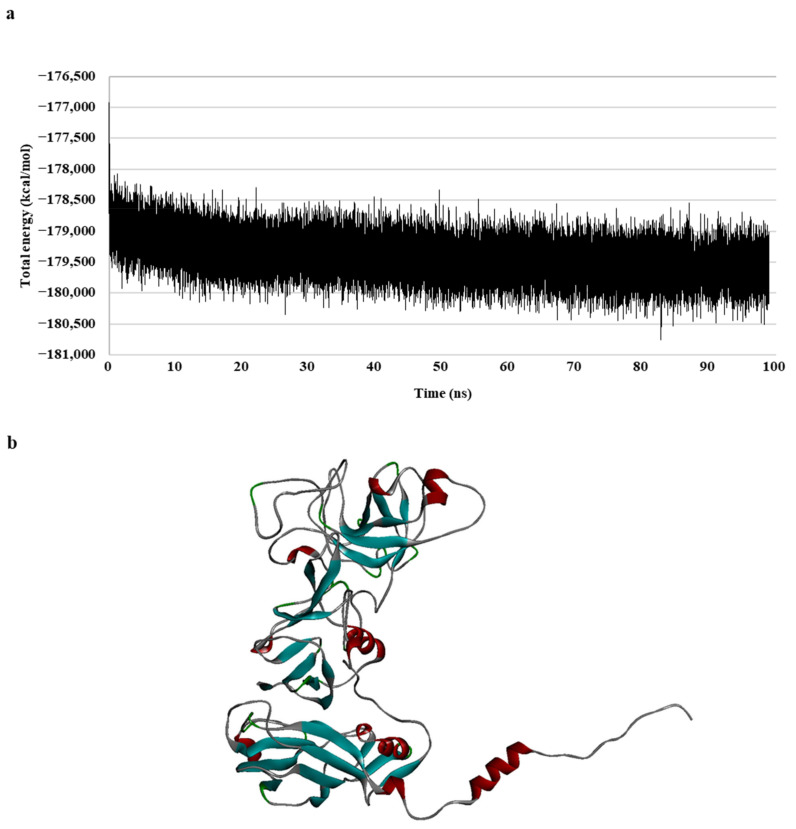
Walrus calicivirus capsid protein homology model. (**a**) Total energy chart of the walrus calicivirus capsid protein homology model during molecular dynamic (MD) simulation and (**b**) 3D structure of the walrus calicivirus capsid protein homology model.

To generate a realistic model of protein dynamic conformational changes, the model was optimized via a 100 ns molecular dynamics (MD) simulation (Figure 1). The MD simulations mimic the physical motion of atoms in protein molecules in a natural environment. Therefore, an MD simulation of the generated homology model was necessary for resolving the structural deficiency of the initially generated structure [31,32]. The total energy of the model remained unchanged for a duration of 30 ns, specifically during the 70–100 ns period (Figure 1a); the result indicated that the model formed a stable conformation after these simulations.

In summary, a 3D model of the walrus calicivirus capsid protein was generated based on two template sequences (with 31.3% identity and 42.7% similarity), and the accuracy, reliability, and stability of the capsid homology model were verified in terms of the total and physical energies of the model. This final model (hereafter, the capsid homology model) was used for further analysis (Figure 1b).

### 2.2. Quality of the Predicted Capsid Homology Model

**Figure 2:**
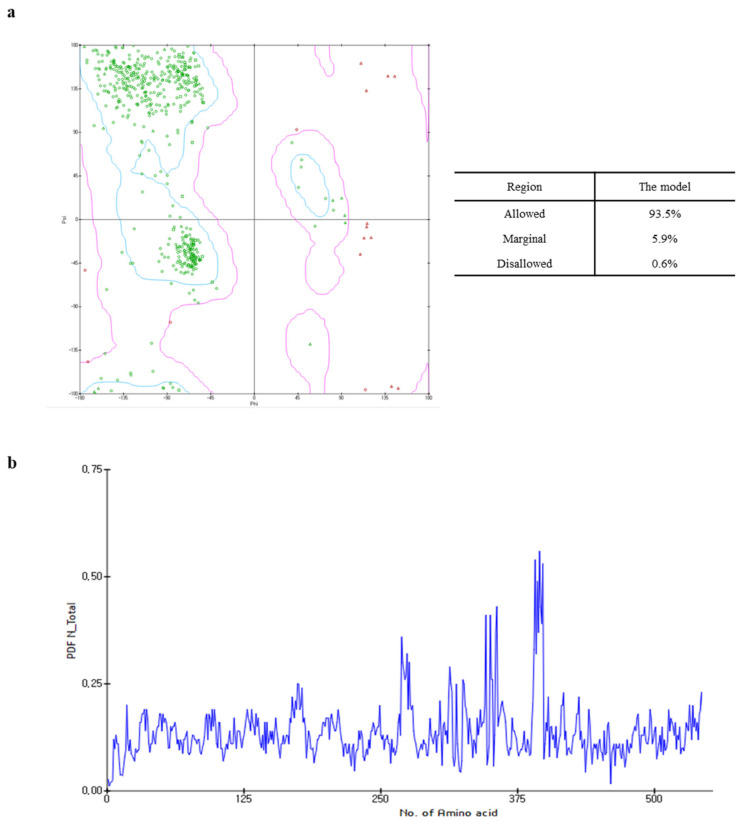
Ramachandran plot (**a**) and probability density function (PDF) total energy plot (**b**) for the selected homology model. In the Ramachandran plot, areas within the blue line are the favored regions, and areas within the purple line are the allowed regions. The red circle, triangle, and square are generously allowed or disallowed amino acid structures.

The Ramachandran plot of the capsid homology model indicated that 93.5% (443 amino acids) of the residues were within the allowed region, whereas 5.9% (28 amino acids) and 0.6% (three amino acids) were in the marginal and disallowed regions, respectively (Figure 2a). Although the PDF total energy of the number 380 amino acid increased, this was excluded from the assessment of model quality as this region did not affect the interaction between the active site and the target ligands to a considerable extent (Figure 2b). Additionally, since the capsid homology model was built using the structural information of FCV as the reference [14], the validation of the capsid homology model was compared with the cryo-electron microscopy structure of FCV by VERIFY-3D, ERRAT, and ProSA. As shown in Figures S3 and S4, the validation analysis of the capsid homology model revealed similar patterns with the cryo-electron microscopy structure of FCV in all validation tools. The validation results suggested that the capsid homology model could be considered reliable, even though the values in the ERRAT and VERIFY-3D tool were lower than the valid standard due to the influence of NTA and loop.

### 2.3. Characterization of Active Sites on the Capsid Homology Model

**Figure 3:**
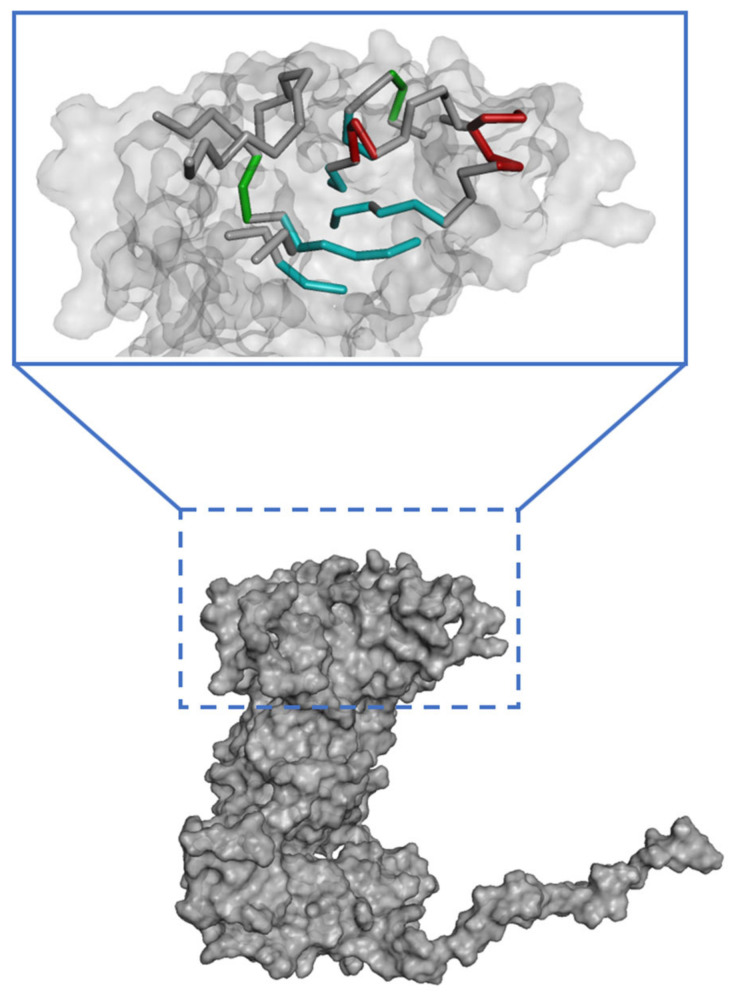
Predicted active site in the walrus calicivirus capsid protein homology model.

**Figure 4:**
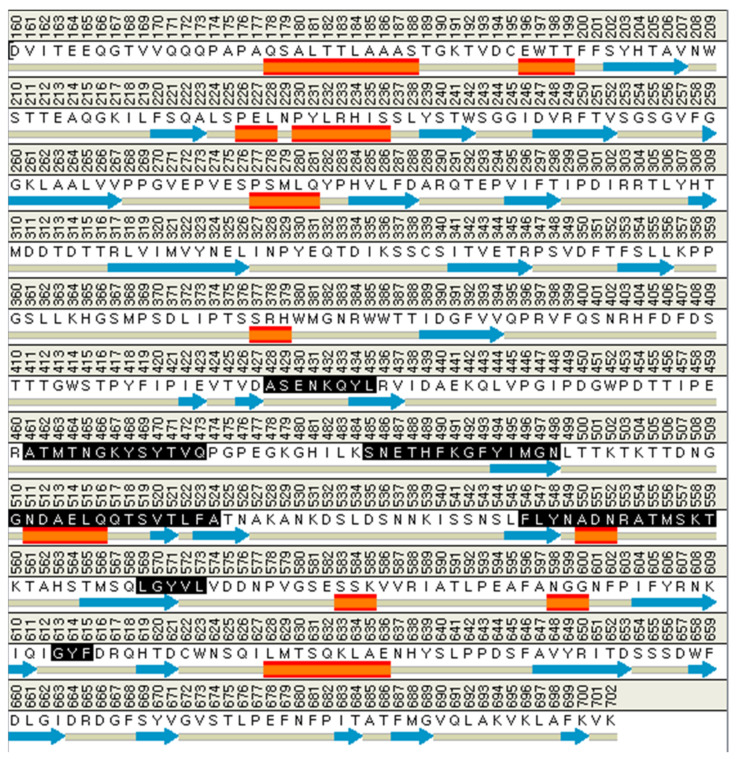
The amino acid composition of the active site in the walrus calicivirus capsid protein homology model (expressed in black). The orange rectangles are alpha helix, and the blue arrows are beta sheet structure.

The binding site spheres of the capsid homology model were explored using an automated “receptor cavities” protocol, which predicts candidate binding sites based on the empty spaces in the structure of the protein. The spheres of the identified candidate binding sites are shown in Figure S5. According to a previous report of the structural characteristics of feline calicivirus, the residues I430, D434, and N495 of the capsid protein are in contact with feline junctional adhesion molecule A, a target molecule for cell entry [14]. Thus, the site comprising I430, D434, and N495 was identified as the most important binding site in the capsid homology model (Figure 3). The putative binding site of the walrus calicivirus capsid protein, as predicted in the generated homology model, comprised the following amino acids: A248, S429, E430, N431, K432, Q433, Y434, L435, A461, T462, M463, T464, N465, G466, K467, Y468, S469, Y470, T471, V472, Q473, S485, N486, E487, T488, H489, F490, K491, G492, F493, Y494, I495, M496, G497, N498, G510, N511, D512, A513, E514, L515, Q516, Q517, T518, S519, V520, T521, L522, F523, A524, F546, L547, Y548, N549, A550, D551, N552, R553, A554, T555, M556, S557, K558, T559, L569, G570, Y571, V572, L573, G613, Y614, and F615 (Figure 4). The binding site is located similarly to the outer VP1 surface of FCV, the region of interaction with fJAM-A.

### 2.4. Molecular Docking Analysis of Phlorotannins on the Capsid Homology Model

To screen potential antiviral candidates from phlorotannins, an in silico analysis of the binding conformations of different phlorotannins to the capsid homology model was performed. The phlorotannins tested in the present study are abbreviated PH1–17. A majority of the examined PHs displayed hydrogen bond electrostatic and/or hydrophobic interactions with the capsid homology model, as summarized in Table S2. PH1, PH5, PH8, PH11, PH12, PH13, PH14, PH15, and PH16 formed over eight predicted hydrogen bonds with the capsid homology model. In particular, PH14 and PH15 formed 10 and 13 hydrogen bonds, as well as five and eight hydrophobic interactions, respectively (Table S2). Weak intermolecular interactions between a ligand and a protein, such as hydrogen bonds and electrostatic and hydrophobic interactions, are key factors in energetically stabilizing a ligand at the surface of a protein’s active site [33,34,35]. The formation of hydrogen bonds between each compound and the viral protein indicates the ability of the compound to recognize and dock to the virus protein [36]. Moreover, the number of hydrophobic atoms in the active site of the protein influences the biological activity of the ligands, and hydrophobic interactions are the most important factors in stabilizing the ligands at the binding interface [34].

**Figure 5:**
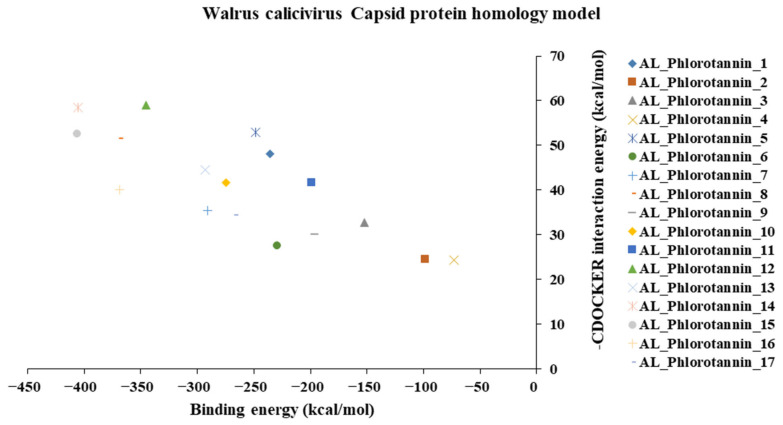
A 2D chart of the docking conformations of phlorotannins in the walrus calicivirus capsid protein homology model. The interactions are expressed as CHARMM-based DOCKER (CDOCKER) interaction energy (kcal/mol) and binding energy (kcal/mol).

The binding and interaction energies of the 17 complexes were compared and analyzed, except for their entropies, which were similar (Table S3 and Figure 5). Compounds with low calculated binding energies and high –Chemistry at Harvard Macromolecular Mechanics (CHARMM)-based DOCKER (–CDOCKER) interaction energies were considered to have high binding affinities for target proteins [37]. The binding energy was between −406.115 and −98.642 kcal/mol, and the –CDOCKER interaction energy ranged from 24.3909 to 58.9415 kcal/mol. The docking score was expressed as a 2D chart using the binding energy (kcal/mol) and –CDOCKER interaction energy (kcal/mol) (Figure 5). Among the various phlorotannins, PH8, PH12, PH14, PH15, and PH16 were characterized as the most favorable candidate ligands, showing relatively high binding affinities with low binding energies and high –CDOCKER interaction energies. These PHs, each possessing more than six phenol rings and 11 hydroxyl groups, formed a stable complex, as each phenol ring and hydroxyl group participated in a bond with the capsid homology model.

**Figure 6:**
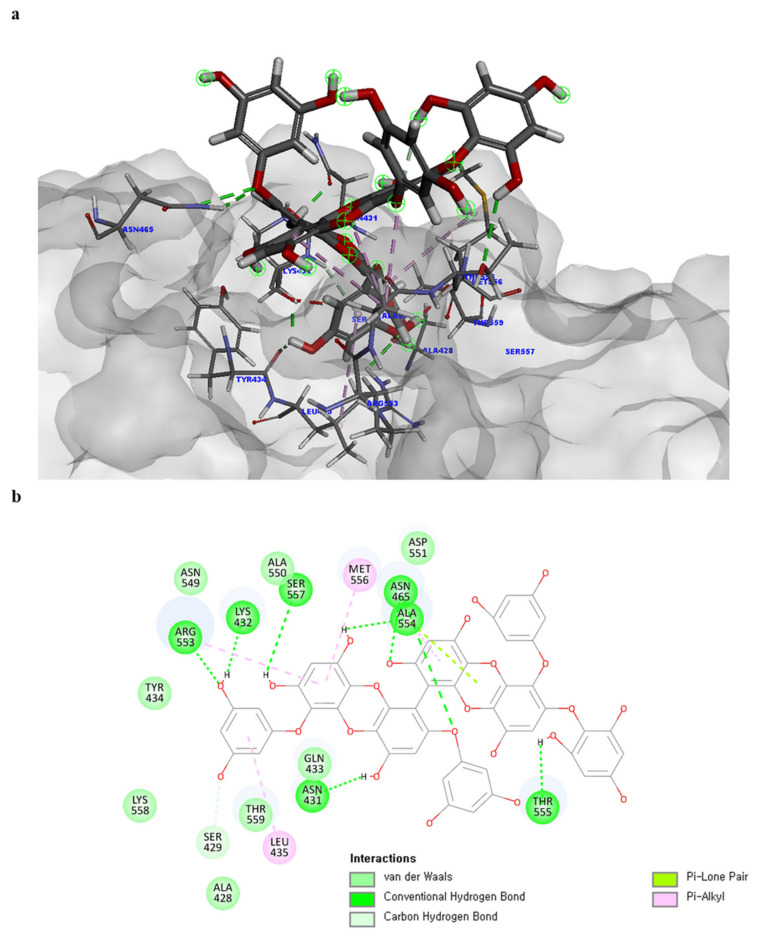
Predicted docking conformation of the PH14-walrus calicivirus capsid protein complex. PH14 is shown as a gray/red stick model. (**a**) PH14 docking to the main amino acids of the walrus calicivirus capsid protein homology model. (**b**) A 2D depiction of the PH14-capsid protein complex.

**Figure 7:**
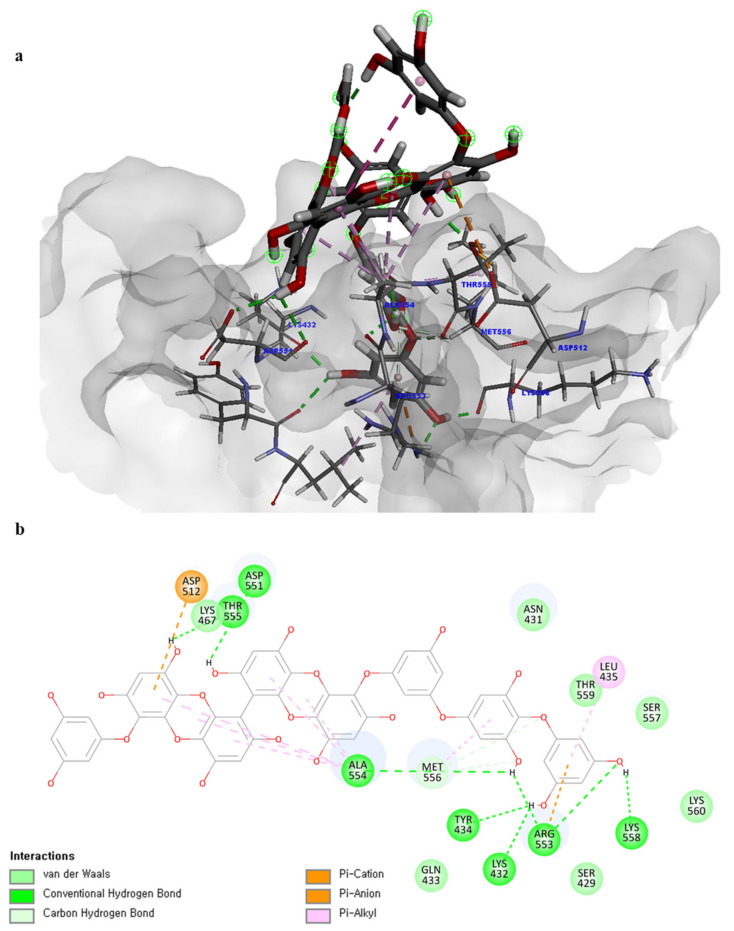
Predicted docking conformation of the PH15-walrus calicivirus capsid protein complex. PH15 is shown as a gray/red stick model. (**a**) PH15 docking to the main amino acids of the walrus calicivirus capsid protein homology model. (**b**) A 2D diagram of the PH15-capsid protein complex.

Of note, PH14 and PH15 showcased the most stable association with the capsid homology model. Although PH14 had a relatively low –CDOCKER energy (3.4846 kcal/mol) owing to its high ligand energy (125.346 kcal/mol), PH14 strongly docked to the capsid homology model with a –CDOCKER interaction energy of 58.4701 kcal/mol and a binding energy of −405.571 kcal/mol (Table S3, Figure 6 and Figure S6a,b). Similarly, PH15 also strongly interacted with the capsid homology model with a –CDOCKER interaction energy of 52.5371 kcal/mol and a binding energy of −406.115 kcal/mol (Table S3, Figure 7 and Figure S6c,d). Both PH14 and PH15 docked in close proximity to the active site of the capsid homology model, displaying favorable hydrogen bond interactions. As shown in the 3D and 2D interaction diagrams of the PH14–capsid protein complex, PH14 interacted in an intricate manner with various amino acids, including S429, N431, K432, L435, N465, R553, A554, T555, M556, and S557. The first, second, and fifth benzene rings of PH14 interacted via four pi bonds with L435, R553, M556, and N465. In addition, the four oxygen atoms in PH14 interacted via hydrogen bonds with S429, R553, N465, and A554 (Figure 6 and Table S4). Regarding PH15, the compound formed 9 pi bonds and 11 hydrogen bonds with various amino acids, including L435 (eight benzene ring), D 512 (second benzene ring), R553 (eight benzene ring), A554 (second, third, and forth benzene ring), and MET556 (seventh benzene ring) (Figure 7 and Table S5). These interactions imply that these phlorotannins can bind stably to the capsid homology model.

Moreover, the binding affinities of the homology model for the receptor molecule fJAM-A were investigated. To confirm the efficacy of PH14 and PH15, we compared and analyzed the binding affinity between fJAM-A and FCV capsid protein (Ligand-free, PDB ID: 6GSI), and fJAM-A and the PH14– and PH15–capsid protein complexes. As shown in Figures S7–S9 and Table S6, both fJAM-A and the PH14– and PH15–capsid protein complexes were predicted to form more stable complexes (−594.64093 kcal/mol and −668.37787 kcal/mol, respectively) than the fJAM-A and FCV capsid protein (−136.51303 kcal/mol). PH14 and PH15 formed interactions with the main amino acids of fJAM-A, including S33, E34, P35, D36, V37, R38, D42, and K46. The results suggest that PH14 and PH15 docked to the capsid homology model and interrupted the complex formation of fJAM-A and capsid protein.

### 2.5. MD Simulation of Phlorotannins on Walrus Calicivirus Capsid Protein

**Figure 8:**
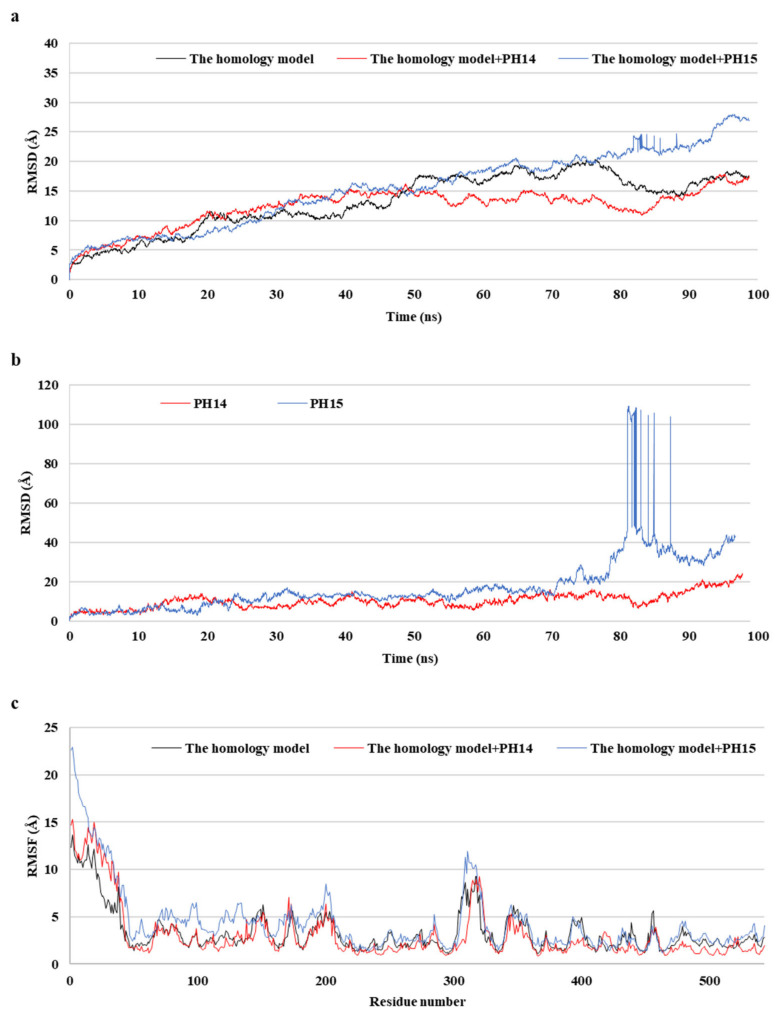
Post-MD structure of the PH14- and PH15-walrus calicivirus capsid protein complex. Root-mean-square deviation (RMSD) for ligand-bound complexes (**a**), RMSD for ligands (**b**), and root-mean-square fluctuation (RMSF) (**c**) of the post-MD structure of the complexes during MD simulation.

**Figure 9:**
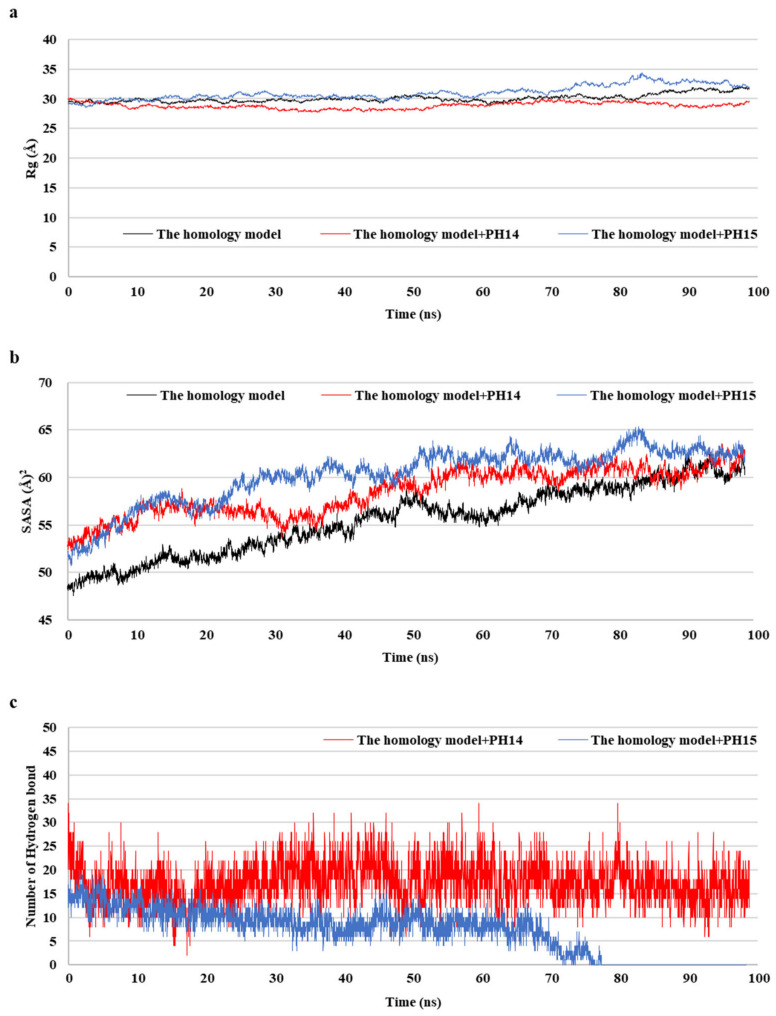
Radius of gyration (Rg) (**a**), solvent-accessible surface area (SASA) (**b**), and number of hydrogen bond (**c**) of the post-MD structure of the complexes during MD simulation.

MD simulations aid in evaluating the stability of ligand–protein complexes [38]. The stability of each of the PH14– and PH15–capsid protein complexes was analyzed by simulating the biological network dynamics for 100 ns; the stabilities were compared with the homology model, a ligand-free structure (Figure 8, Figure 9 and Figure S10). The two phlorotannin–capsid homology model complexes showed similar root-mean-square deviation (RMSD) of the initial frame in the trajectory with the homology model until about 30 ns. Afterward, the PH14–capsid protein complex retained a pattern similar with the homology model until 100 ns, while the PH15–capsid protein complex showed increasing RMSD. Also, PH14 generally showed lower ligand RMSD than PH15 until about 70 ns, afterward, PH15 showed the rapidly increasing ligand RMSD. The lower ligand RMSD of PH14 suggested the higher stability of the PH14-capsid protein complex than the PH15-capsid protein complex. Furthermore, the root-mean-square fluctuation (RMSF) of most amino acid residues of the PH15–capsid protein complex was higher than those of the homology model and PH14–capsid protein complex (Figure 8). In addition, the PH14–capsid protein complex maintained a lower radius of gyration (Rg) than that of the homology model. Also, the PH14–capsid protein complex showed generally lower solvent-accessible surface area (SASA) values than PH15–capsid protein complex. Furthermore, the PH14–capsid protein complex maintained a certain number of hydrogen bonds during the simulation, whereas the PH15–capsid protein complex lost the hydrogen bonds at 78 ns. Finally, PH14 remained continuously docked to the active site of the capsid homology model at the end of the simulation (Figure S11). The results of the interaction analysis indicated that PH14 achieved a stable conformation with the capsid homology model, confirming the integrity of the complex over the simulation trajectory. In particular, the hydrogen bond interactions between PH14 and certain amino acids, including A428, A550, T555, and S557, were essential for stabilizing the complex by sustaining the interaction during simulations (Figure S12 and Table S7). The frequencies of the interactions between PH14 and A550, S557 (two bonds), A428, and R555 were 0.85, 0.59, 0.58, 0.56, and 0.42, respectively (Table S7). These MD simulation results indicated that PH14 could form strong and stable complexes with the capsid protein homology model and could therefore be potent inhibitors of the walrus calicivirus.

## 3. Discussion

The recent pandemic has led to a rapid growth in drug development technologies for creating better systems to meet both present and future global health challenges [39,40]. In silico screening is one of the most important strategies applied in the field of candidate discovery to expedite the screening steps. Numerous in silico-based studies on targets have already been published [2,38,41,42,43,44], and the studies or results can serve as a foundation for exploring potential candidates and experimental validation. In addition, with improvement in computational technologies, virtual screening has become an important tool for exploring drug candidates against a new virus, even when the crystal structures of the viral proteins are unknown [2,45].

In the present study, a monomer VP1 capsid protein structure was used in the homology modeling, docking, and molecular dynamics studies. Since the phlorotannin binding site is located at the exposed outer VP1 surface, at its interface with the host anchor molecule, the usage of a monomer VP1 is a strategy to explore the inhibitor by simplifying the operation of escape the virus. As a result, phlorotannins, including 2,7-phloroglucinol-6,6-bieckol, were predicted as potential candidate inhibitors of a novel calicivirus isolated from walrus. However, further studies on inhibition by PHs in oligomeric forms involving several molecules of VP1 and VP2 should be performed to evaluate how PHs acts on the whole structure of the capsid.

Moreover, the present study demonstrated that in silico-based drug discovery approaches, such as homology modeling, molecular docking, and MD simulations can be valuable for accelerating the discovery of candidate inhibitor compounds against novel potential viral pathogens, whose 3D structures have not been validated experimentally.

Marine natural products, such as phlorotannins, are secondary metabolites with much more diverse and unique structures than those found in terrestrial organisms. Although phlorotannins have been demonstrated to have various effects in previous research, they are still less studied than natural products of land origin. This indicates that the prediction of absorption, distribution, metabolism, excretion, and toxicity of marine natural products can be challenging, owing to the limited information available for an artificial intelligence-based training set. However, marine natural products demonstrate promising potential as inhibitors of proteins related to various diseases. Therefore, it is necessary to utilize marine natural products in studies focusing on safeguarding human health effectively against current as well as future infectious diseases.

Analysis of the 3D structure of marine viral protein is more challenging than that of terrestrial organisms due to the difficulty of obtaining marine animal samples. Therefore, although recent studies have revealed the amino acid sequences of several proteins, the actual 3D structures of most marine viruses are still unknown. This lack of 3D structural data precludes in vitro and in vivo research required to identify effective inhibitors. To address this limitation, researchers should focus on developing computational modeling tools for generating reliable protein tertiary structure models based solely on amino acid sequence information. The prediction of viral protein structures and high-throughput screening of multiple candidate inhibitor compounds, including natural products are key strategies for responding to new virus outbreaks, including any unknown pathogens. Furthermore, in a future where the analytical experiments become possible, additional investigations are required to confirm the capsid protein inhibition by PHs. Additionally, further studies on inhibition of FCV by PHs are required based on this in silico screening study.

## 4. Materials and Methods

### 4.1. Retrieval of the Protein Sequence and Homology Modeling

The full amino acid sequence of the walrus calicivirus capsid protein was obtained from the National Center for Biotechnology Information (Reference Sequence: NP_777371.1) and UniProt databases (Q9DKW1), accessed on September 2022. Its 3D structure was then constructed, based on the available template deposited in the RCSB Protein Data Bank (PDB). Two high-resolution templates (PDB IDs 2GH8 and 6GSI) were downloaded from the PDB database. Homology modeling was performed using Discovery Studio 2022 (Biovia, San Diego, CA, USA). The top protein 3D structure was selected based on the PDF total energy, PDF physical energy, and DOPE score values. The PDF total energy was calculated using the sum of all homology-derived and stereochemical pseudo-energy terms. Its means of satisfaction of spatial restraints were generated during the model construction [46]. The PDF physical energy, i.e., the sum of stereochemical pseudo-energy terms, is a value related to the physical properties such as bond, angle, and torsion angle among PDF energies. DOPE is an atomic distance-based statistical potential; the DOPE score of a protein represents the conformational energy that measures the relative stability of one molecular conformation with respect to other conformations of the same protein. It aids in selecting the best model out of the predicted model structures [46].

### 4.2. MD Simulation

To investigate the dynamic behavior of the walrus calicivirus capsid protein homology model, MD simulation was performed using the CHARMM force field of Discovery Studio 2022. Each step of the MD process was conducted according to the following simulation protocols [41,42]: solvation with explicit periodic boundary (orthorhombic box); 1000 steps of minimization using the steepest descent algorithm to resolve any initial poor contacts within the system, without creating large distortions in the overall structure; 2000 steps of minimization using adopted basis Newton–Raphson algorithm to remove the constraints used in the first minimization step or changes made to different types of constraints to achieve different goals of the minimization; 100 ps heating at 300 K; 500 ps equilibration; 500 ps production in standard number of particles, volume, and temperature ensemble; and 20 ns nanoscale MD in a standard number of particles, pressure, and temperature ensemble to identify the backbone and side-chain adjustments of the receptor concerning the ligand and to analyze the contribution of H-bonding, hydrophobic contacts, and polar and Van der Waals interactions in maintaining the stability of the receptor cavity in a dynamic condition. The simulation was performed at a time step of 2 fs, and the trajectory frame was collected every 2 ps. The stability of each system was assessed by computing the RMSD and fluctuation over the entire simulation period.

### 4.3. Model Validation and Binding Site Refinement

The refined model was evaluated using a Ramachandran plot, the PDF total and PDF physical energy, as well as the DOPE score. Further, the validation of the capsid homology model was confirmed by VERIFY-3D, ERRAT, and ProSA tools. The binding site of the walrus calicivirus capsid protein homology model was defined from the receptor cavities, and the site composed of the main amino acids was selected based on data from the existing literature [14].

### 4.4. 3D Structure of Phlorotannins

The 3D structures of 17 phlorotannins were obtained from PubChem or produced using the drawing tool of Discovery Studio 2022 (Biovia). The compound names and PubChem compound ID (CID) numbers are as follows: triphloroethol-A (CID: 21626545), phloroglucinol (CID: 359), eckol (CID: 145937), dioxinodehydroeckol (CID: 10429214), 2-phloroeckol (CID: 5320532), 7-phloroeckol (CID: 10480940), fucodiphloroethol-G (CID: 44590821), dieckol (CID: 3008868), fucofuroeckol-A (CID: 23426721), phlorofucofuroeckol-A (CID: 130976), phlorofucofuroeckol-B (CID: 15984097), 6,6′-bieckol (CID: 137388), 8,8′-bieckol (CID: 3008867), 2,7-phloroglucinol-6,6-bieckol (drew), pyrogallol-phloroglucinol-6,6-bieckol (drew), 2-O-(2,4,6-trihydroxyphenyl)-6,6′-bieckol (CID: 16132364), and diphlorethohydroxycarmalol (CID: 16075395). Geometry optimization of the phlorotannin 3D structures was performed using the energy minimization protocol of Discovery Studio 2022, and the phlorotannins were numbered in ascending order from PH1 to PH17 (Table 1).

### 4.5. Molecular Docking Analysis between the Walrus Calicivirus Capsid Protein and PHs

Molecular docking analysis was performed to assess the respective binding positions and energies of the different PHs to the constructed walrus calicivirus capsid protein homology model. For this analysis, flexible docking based on the CHARMM and Calculate Binding Energies tools in Discovery Studio 2022 (Biovia) was used. The area from the center of the active site within a radius of 17.8 Å (considering the size of PHs) was designated as the binding pocket of the capsid protein. The binding site and ligand were allowed to move freely during the docking simulation. To screen candidate compounds, the PH–protein complexes for each molecule were subsequently examined by comparing entropy and energy. The docking positions of the selected PHs on the walrus calicivirus capsid protein were expressed as 2D diagrams and 3D crystal structures.

## Figures and Tables

**Table 1:** Phlorotannins used as docking candidates for the walrus calicivirus capsid protein homology model.

PH No.	Phlorotannins	Structure	CID	MW (Da)
PH1	Triphloroethol-A	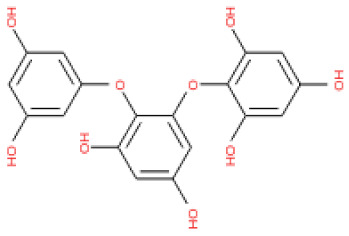	21626545	374.31
PH2	Phloroglucinol	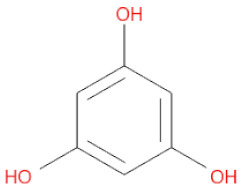	359	126.11
PH3	Eckol	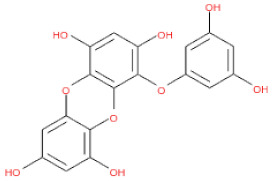	145937	372.29
PH4	Dioxinodehydroeckol	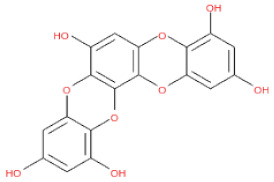	10429214	370.28
PH5	2-phloroeckol	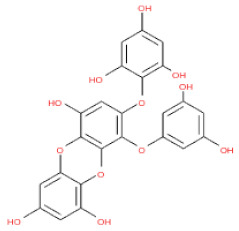	5320532	496.39
PH6	7-phloroeckol	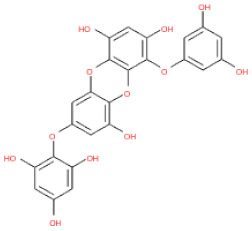	10480940	496.39
PH7	Fucodiphloroethol-G	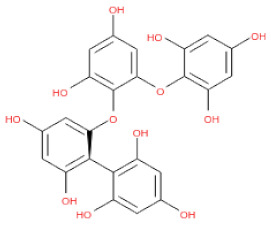	44590821	498.41
PH8	Dieckol	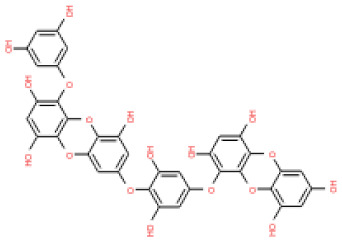	3008868	742.57
PH9	Fucofuroeckol-A	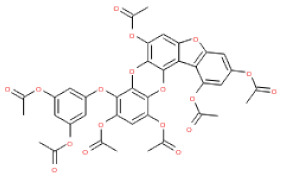	23426721	772.64
PH10	Phlorofucofuroeckol-A	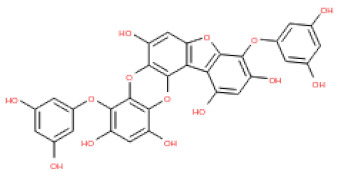	130976	602.47
PH11	Phlorofucofuroeckol-B	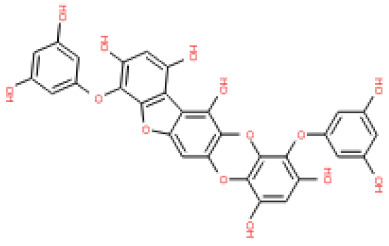	15984097	602.47
PH12	6,6′-bieckol	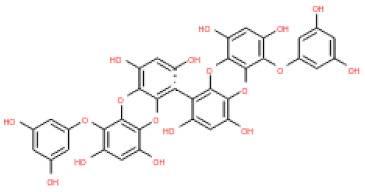	137388	742.57
PH13	8,8′-Bieckol	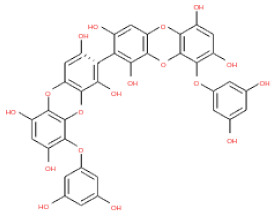	3008867	742.57
PH14	2,7-phloroglucinol-6,6-bieckol	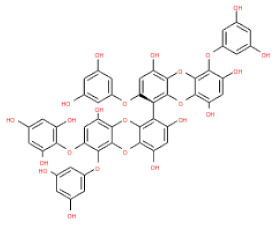	drew	974.77
PH15	Pyrogallol-phloroglucinol-6,6-bieckol	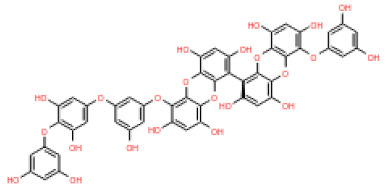	drew	974.77
PH16	2-O-(2,4,6-trihydroxyphenyl)-6,6′-bieckol	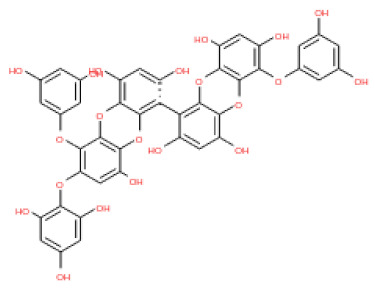	16132364	866.67
PH17	Diphlorethohydroxycarmalol	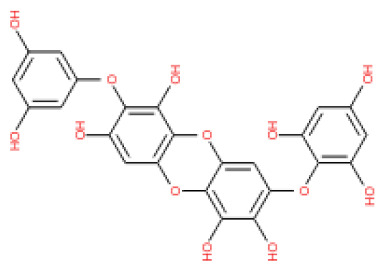	16075395	512.39

CID: compound ID; MW: molecular weight.

## Data Availability

Data is contained within the article or supplementary materials.

## Supplementary Materials

The following supporting information can be downloaded at https://www.mdpi.com/article/10.3390/ijms242115774/s1.
