# Supplementary material for: Structure-Based In Silico Screening of Marine Phlorotannins for Potential Walrus Calicivirus Inhibitor
Source: Int J Mol Sci. 2023 Oct 30;24(21):15774. doi: 10.3390/ijms242115774 (PMC10647355; doi:10.3390/ijms242115774)
Supplement: Supplementary file 1 [file ijms-24-15774-s001.zip › supplementary figures.pptx]

## Slide 1
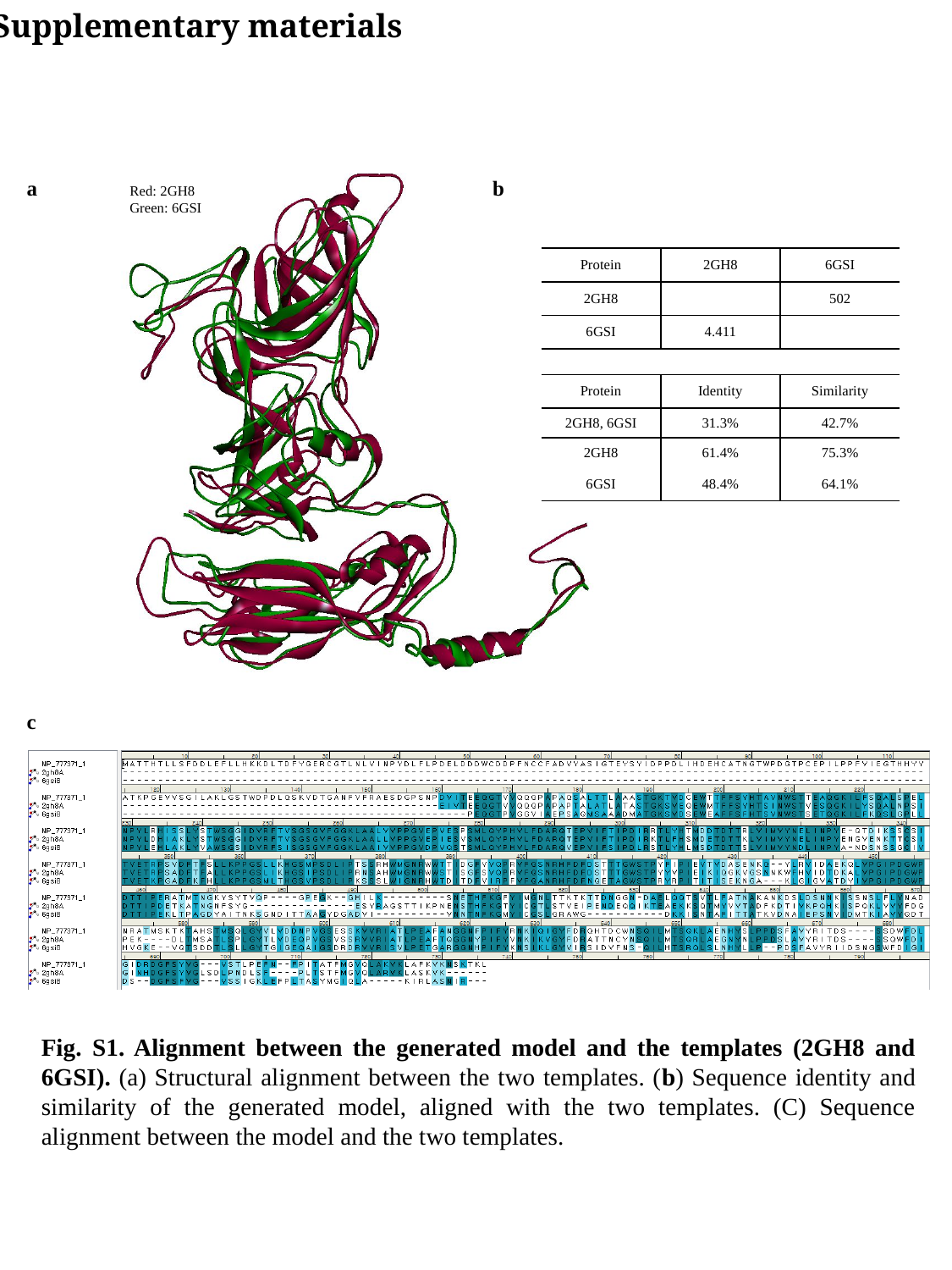

Supplementary materials
a
b
Red: 2GH8
Green: 6GSI
| Protein | 2GH8 | 6GSI |
| --- | --- | --- |
| 2GH8 | | 502 |
| 6GSI | 4.411 | |
| Protein | Identity | Similarity |
| --- | --- | --- |
| 2GH8, 6GSI | 31.3% | 42.7% |
| 2GH8 | 61.4% | 75.3% |
| 6GSI | 48.4% | 64.1% |
c
Fig. S1. Alignment between the generated model and the templates (2GH8 and 6GSI). (a) Structural alignment between the two templates. (b) Sequence identity and similarity of the generated model, aligned with the two templates. (C) Sequence alignment between the model and the two templates.

## Slide 2
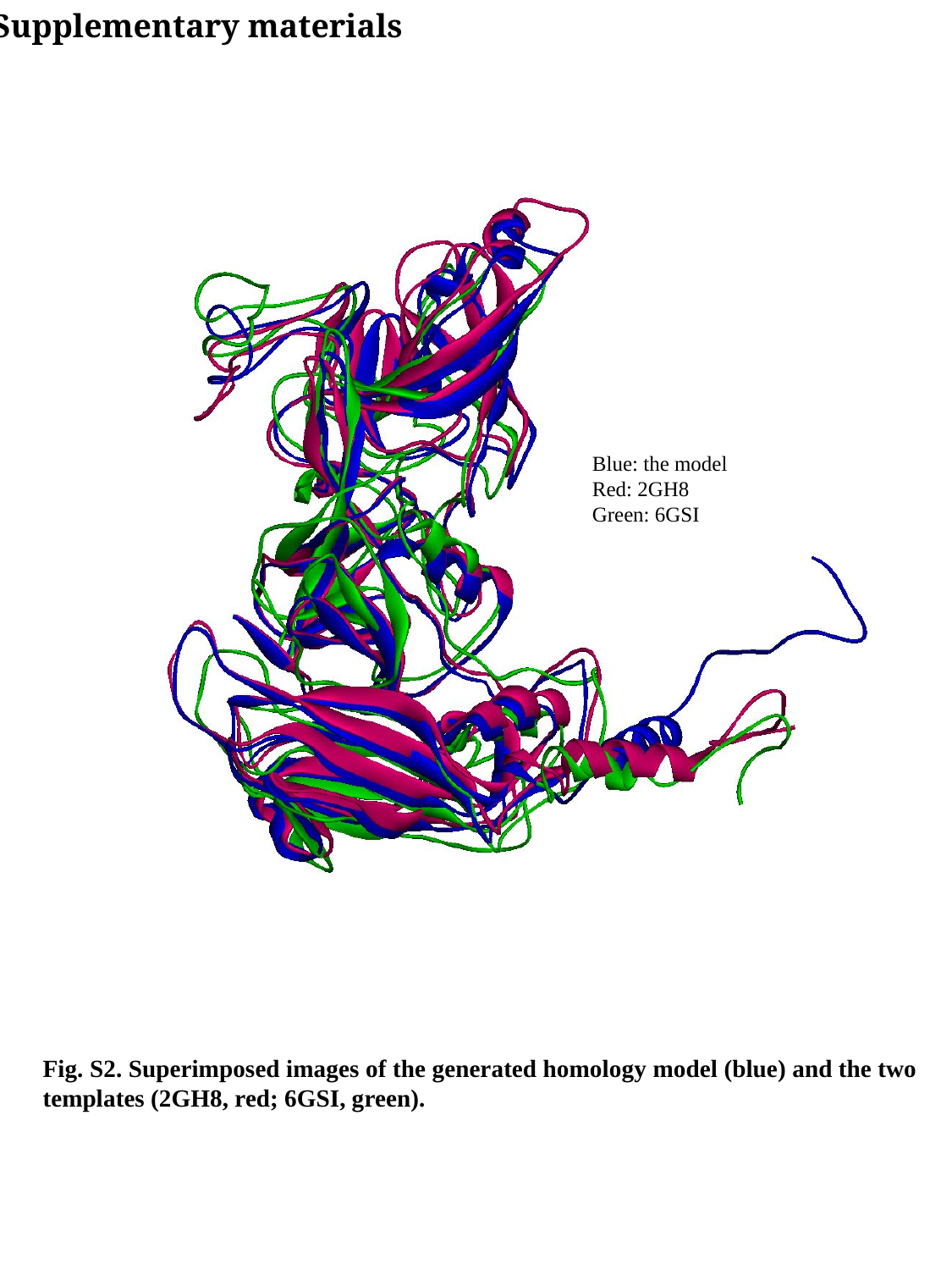

Supplementary materials
Blue: the model
Red: 2GH8
Green: 6GSI
Fig. S2. Superimposed images of the generated homology model (blue) and the two templates (2GH8, red; 6GSI, green).

## Slide 3
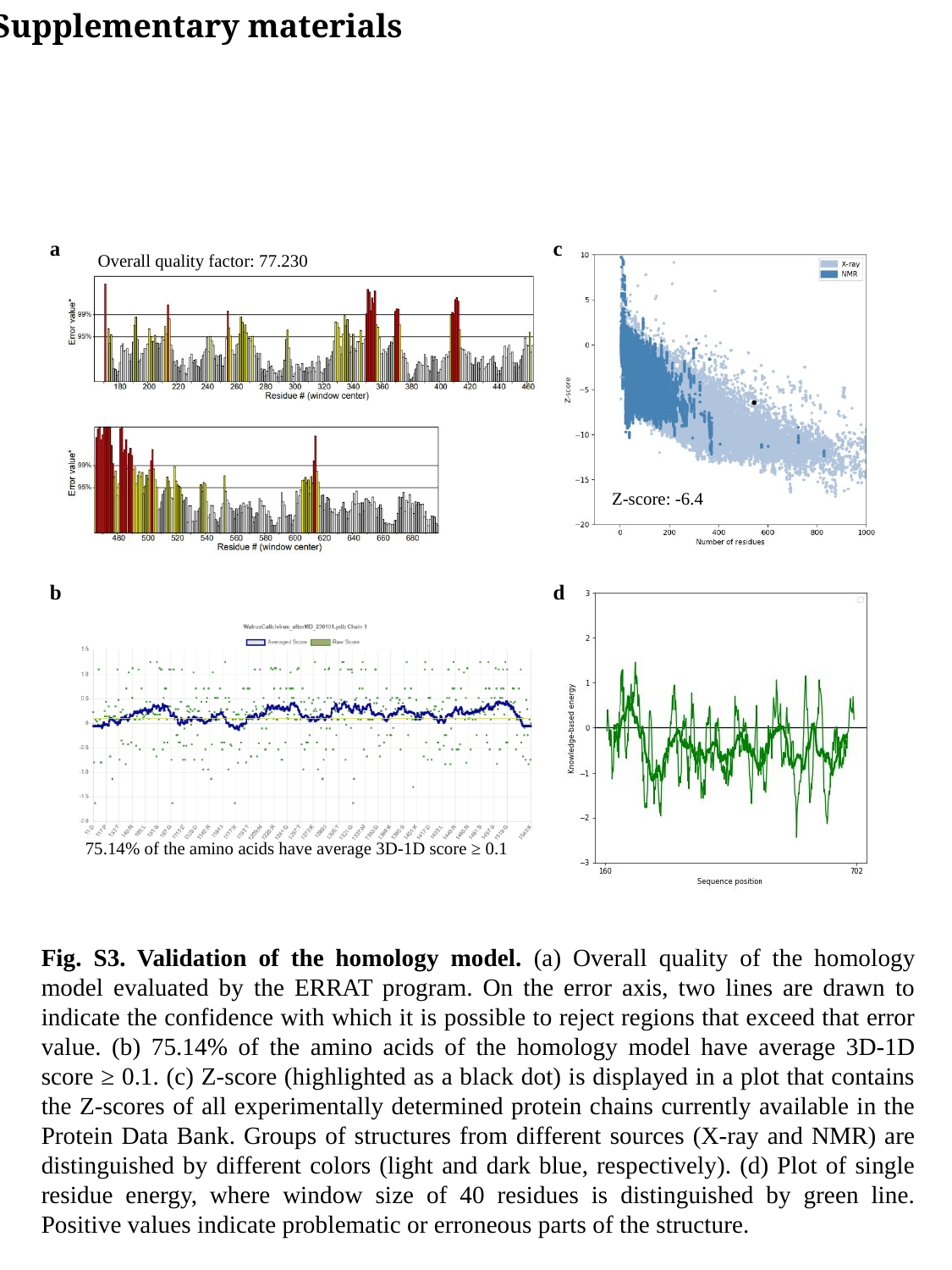

Supplementary materials
a
c
Overall quality factor: 77.230
Z-score: -6.4
b
d
75.14% of the amino acids have average 3D-1D score ≥ 0.1
Fig. S3. Validation of the homology model. (a) Overall quality of the homology model evaluated by the ERRAT program. On the error axis, two lines are drawn to indicate the confidence with which it is possible to reject regions that exceed that error value. (b) 75.14% of the amino acids of the homology model have average 3D-1D score ≥ 0.1. (c) Z-score (highlighted as a black dot) is displayed in a plot that contains the Z-scores of all experimentally determined protein chains currently available in the Protein Data Bank. Groups of structures from different sources (X-ray and NMR) are distinguished by different colors (light and dark blue, respectively). (d) Plot of single residue energy, where window size of 40 residues is distinguished by green line. Positive values indicate problematic or erroneous parts of the structure.

## Slide 4
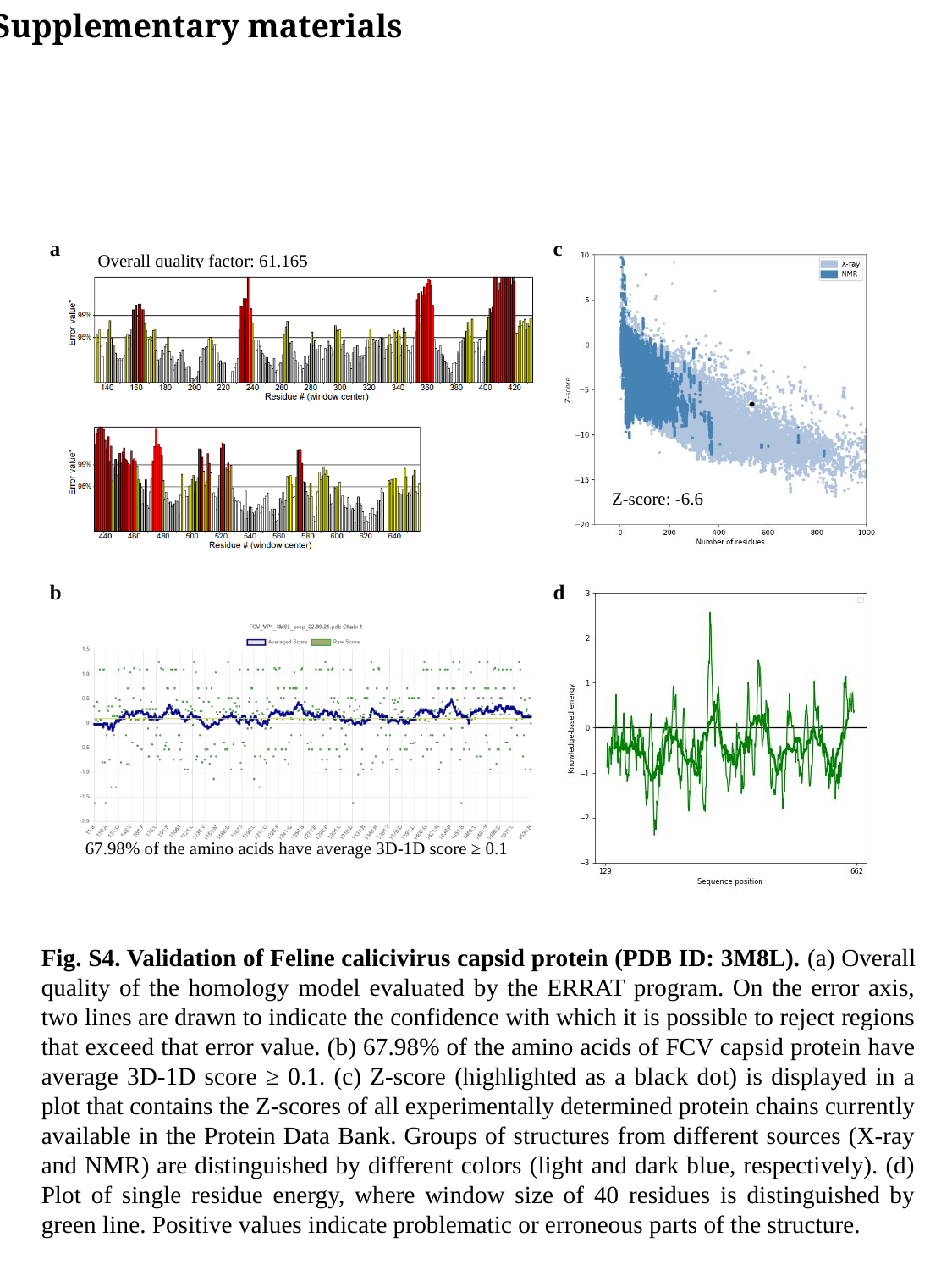

Supplementary materials
a
c
Overall quality factor: 61.165
Z-score: -6.6
b
d
67.98% of the amino acids have average 3D-1D score ≥ 0.1
Fig. S4. Validation of Feline calicivirus capsid protein (PDB ID: 3M8L). (a) Overall quality of the homology model evaluated by the ERRAT program. On the error axis, two lines are drawn to indicate the confidence with which it is possible to reject regions that exceed that error value. (b) 67.98% of the amino acids of FCV capsid protein have average 3D-1D score ≥ 0.1. (c) Z-score (highlighted as a black dot) is displayed in a plot that contains the Z-scores of all experimentally determined protein chains currently available in the Protein Data Bank. Groups of structures from different sources (X-ray and NMR) are distinguished by different colors (light and dark blue, respectively). (d) Plot of single residue energy, where window size of 40 residues is distinguished by green line. Positive values indicate problematic or erroneous parts of the structure.

## Slide 5
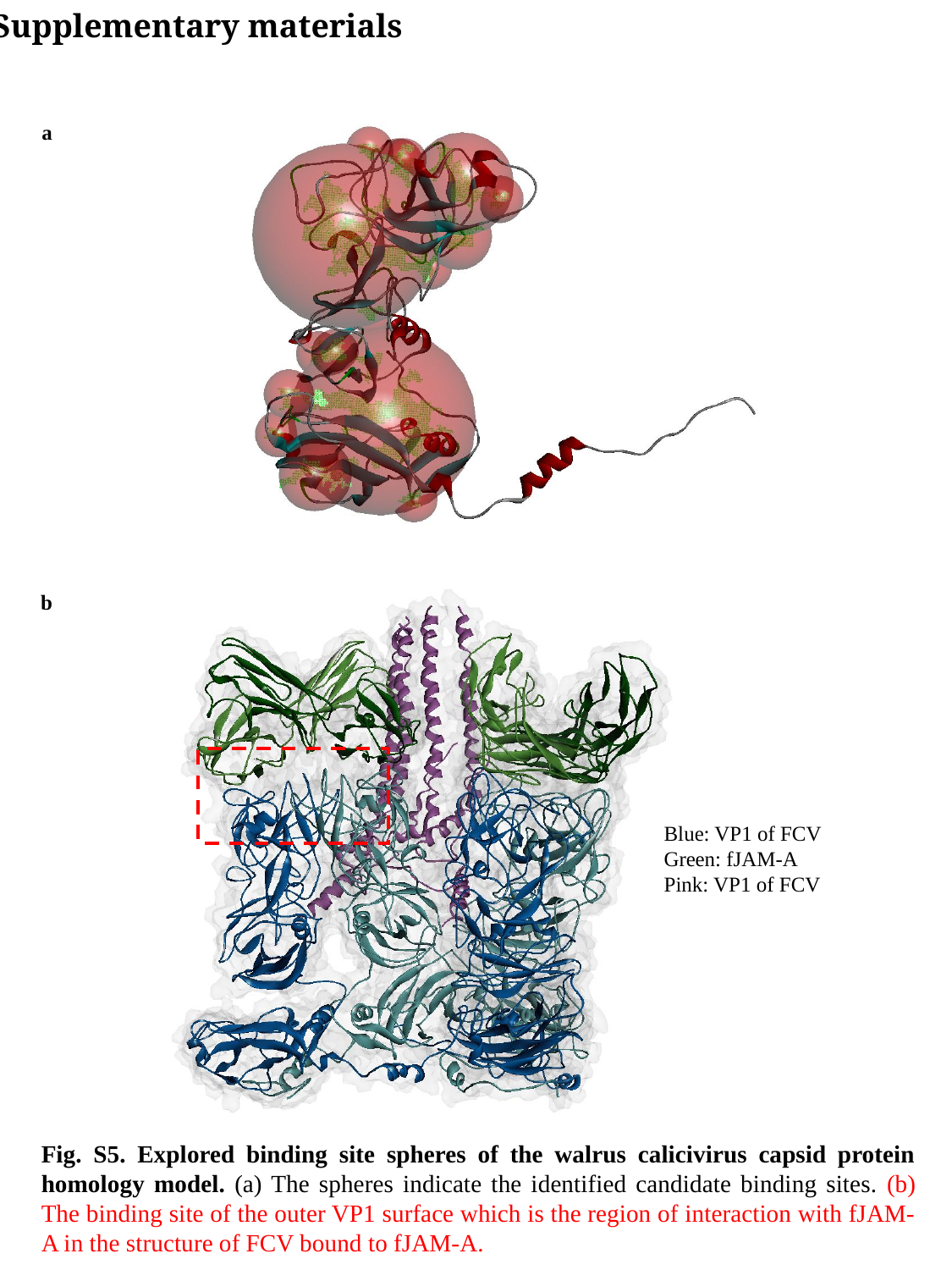

Supplementary materials
a
b
Blue: VP1 of FCV
Green: fJAM-A
Pink: VP1 of FCV
Fig. S5. Explored binding site spheres of the walrus calicivirus capsid protein homology model. (a) The spheres indicate the identified candidate binding sites. (b) The binding site of the outer VP1 surface which is the region of interaction with fJAM-A in the structure of FCV bound to fJAM-A.

## Slide 6
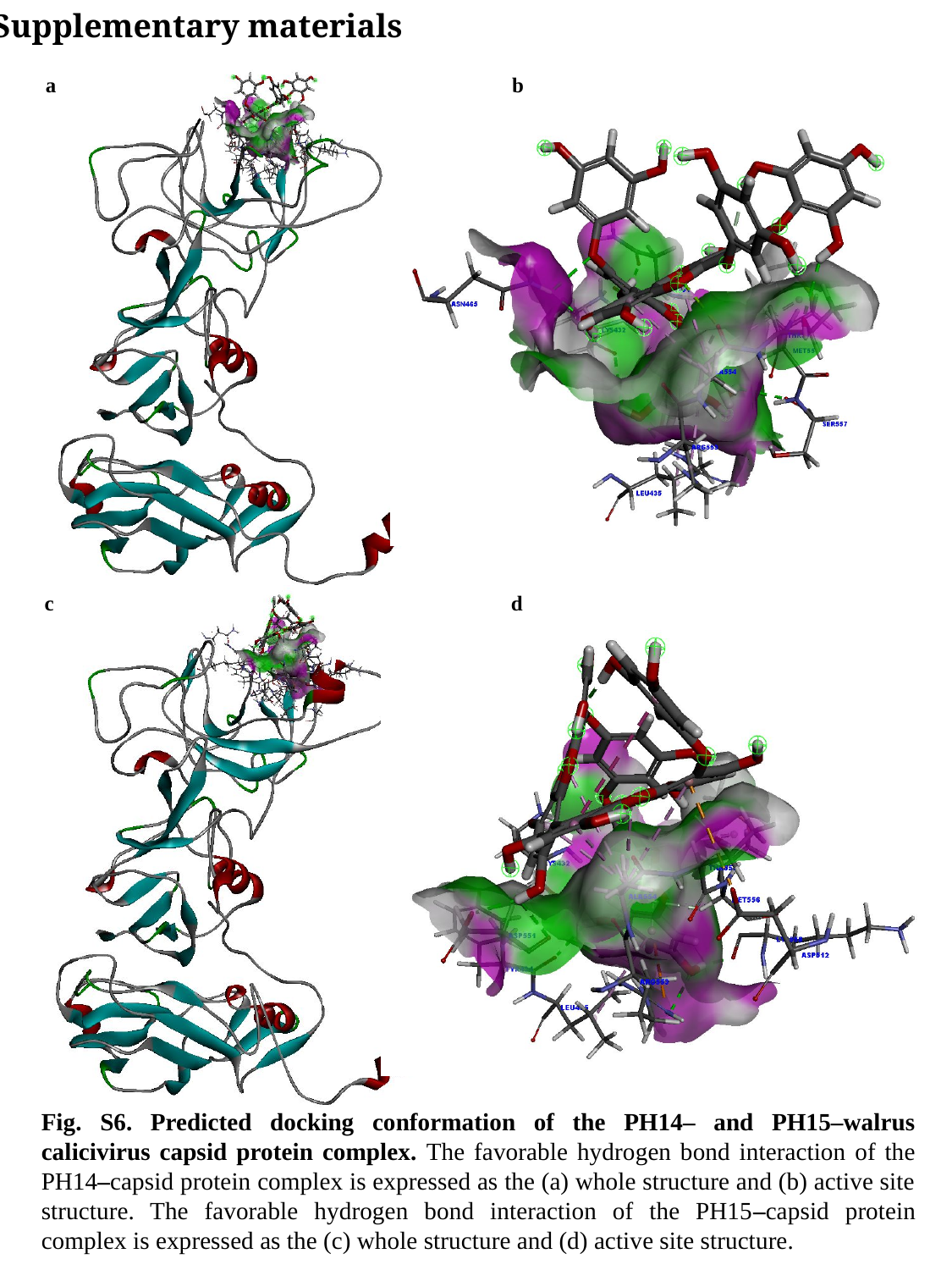

Supplementary materials
a
b
c
d
Fig. S6. Predicted docking conformation of the PH14– and PH15–walrus calicivirus capsid protein complex. The favorable hydrogen bond interaction of the PH14–capsid protein complex is expressed as the (a) whole structure and (b) active site structure. The favorable hydrogen bond interaction of the PH15–capsid protein complex is expressed as the (c) whole structure and (d) active site structure.

## Slide 7
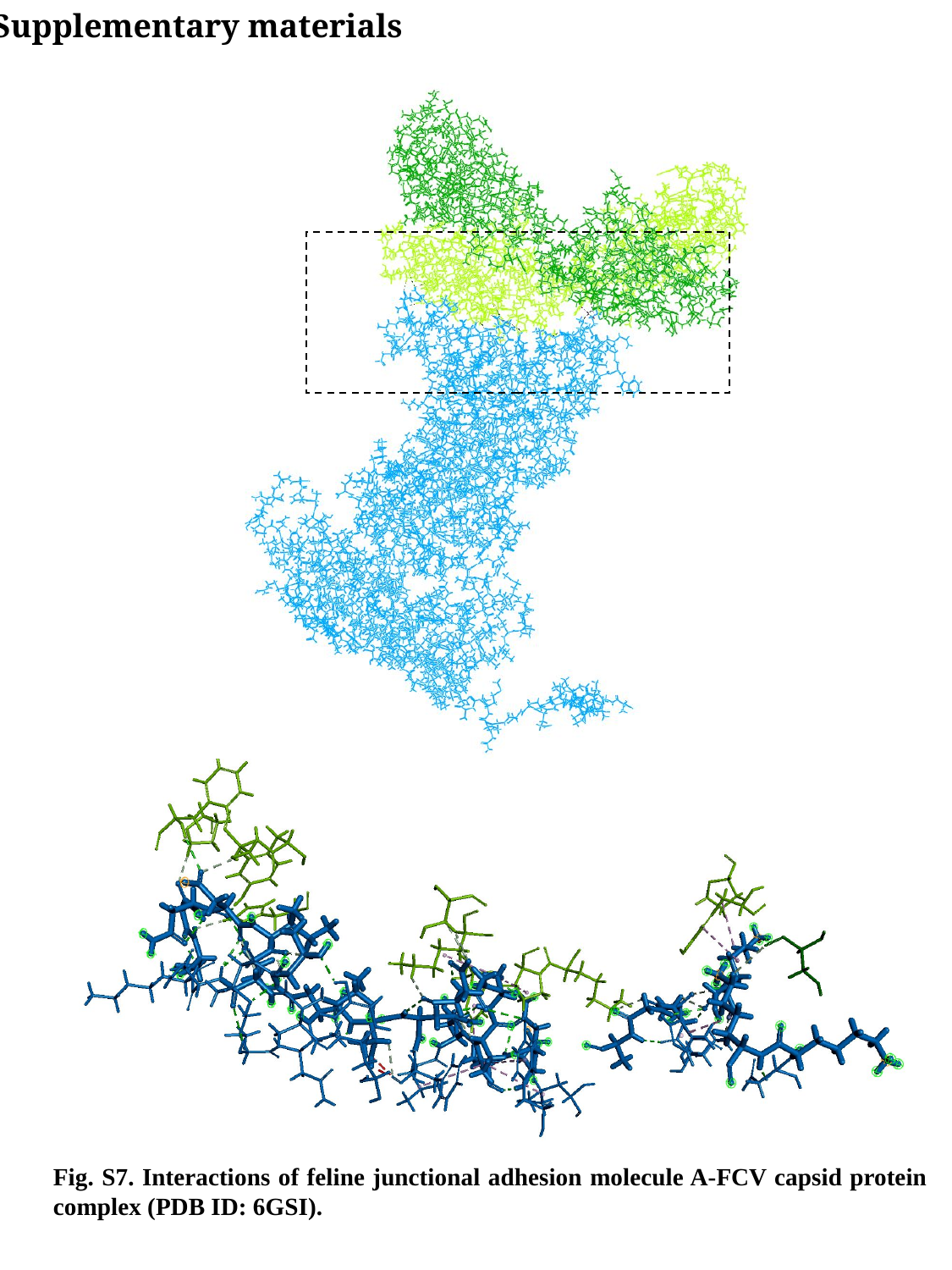

Supplementary materials
Fig. S7. Interactions of feline junctional adhesion molecule A-FCV capsid protein complex (PDB ID: 6GSI).

## Slide 8
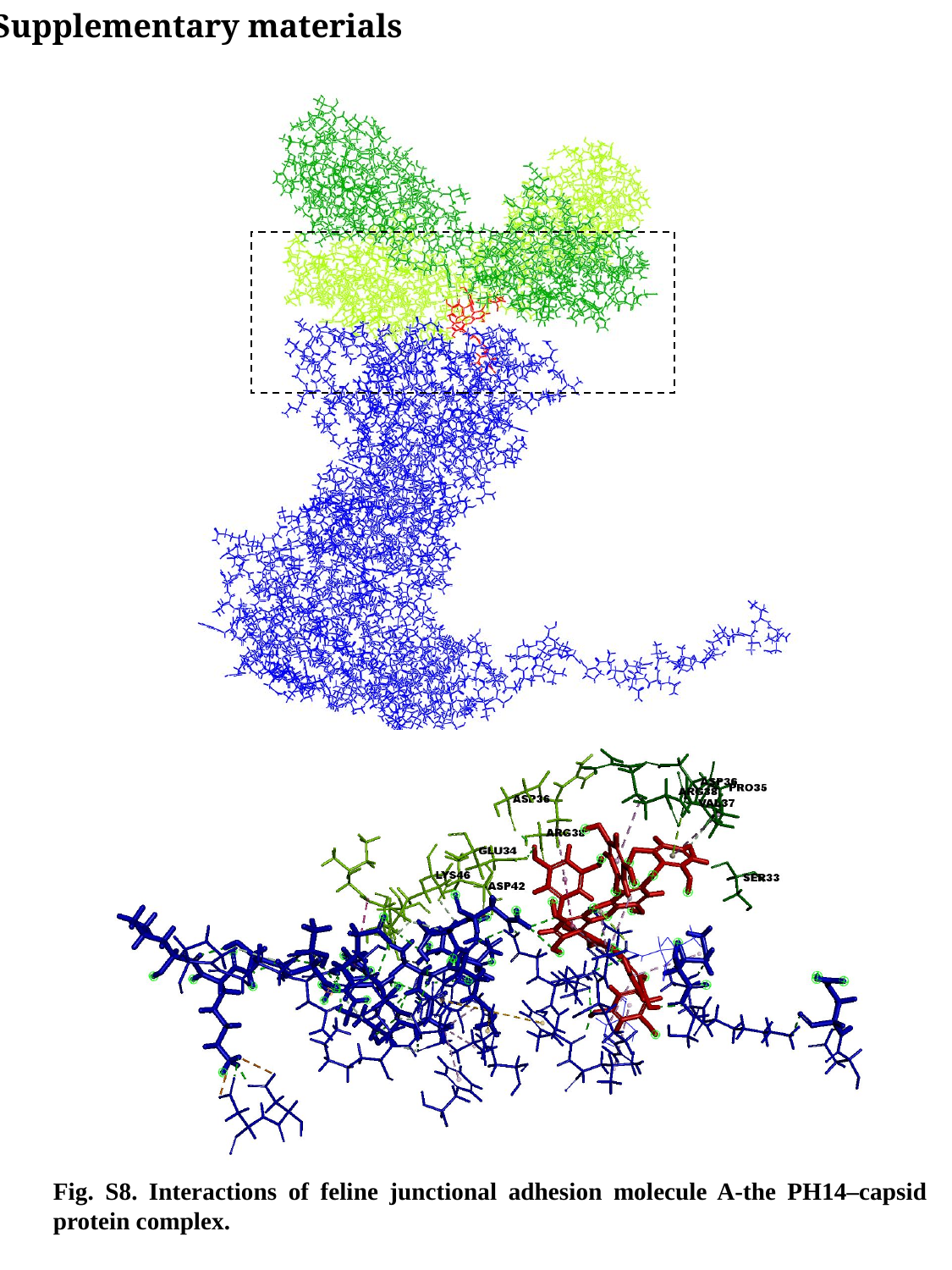

Supplementary materials
Fig. S8. Interactions of feline junctional adhesion molecule A-the PH14–capsid protein complex.

## Slide 9
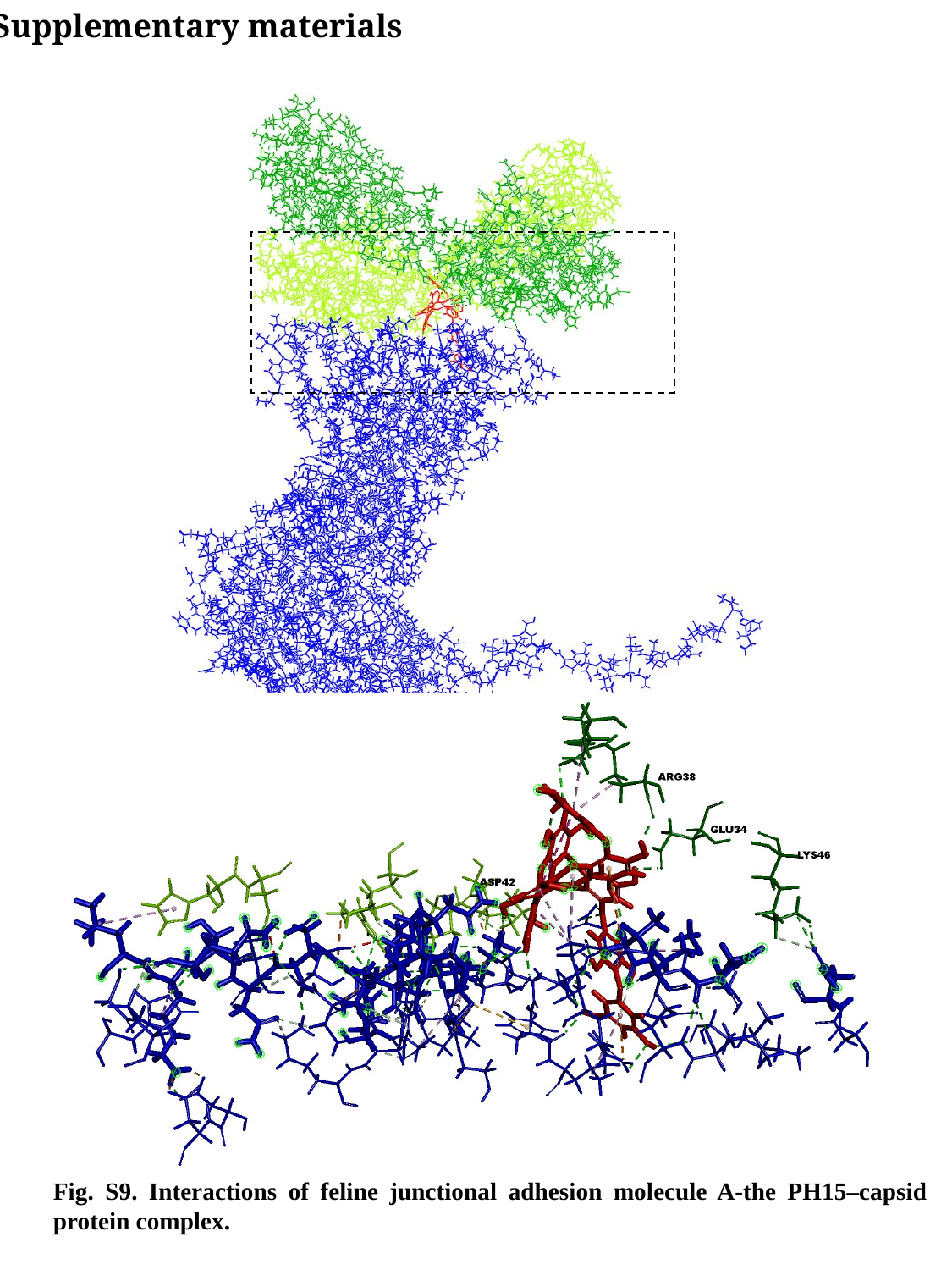

Supplementary materials
Fig. S9. Interactions of feline junctional adhesion molecule A-the PH15–capsid protein complex.

## Slide 10
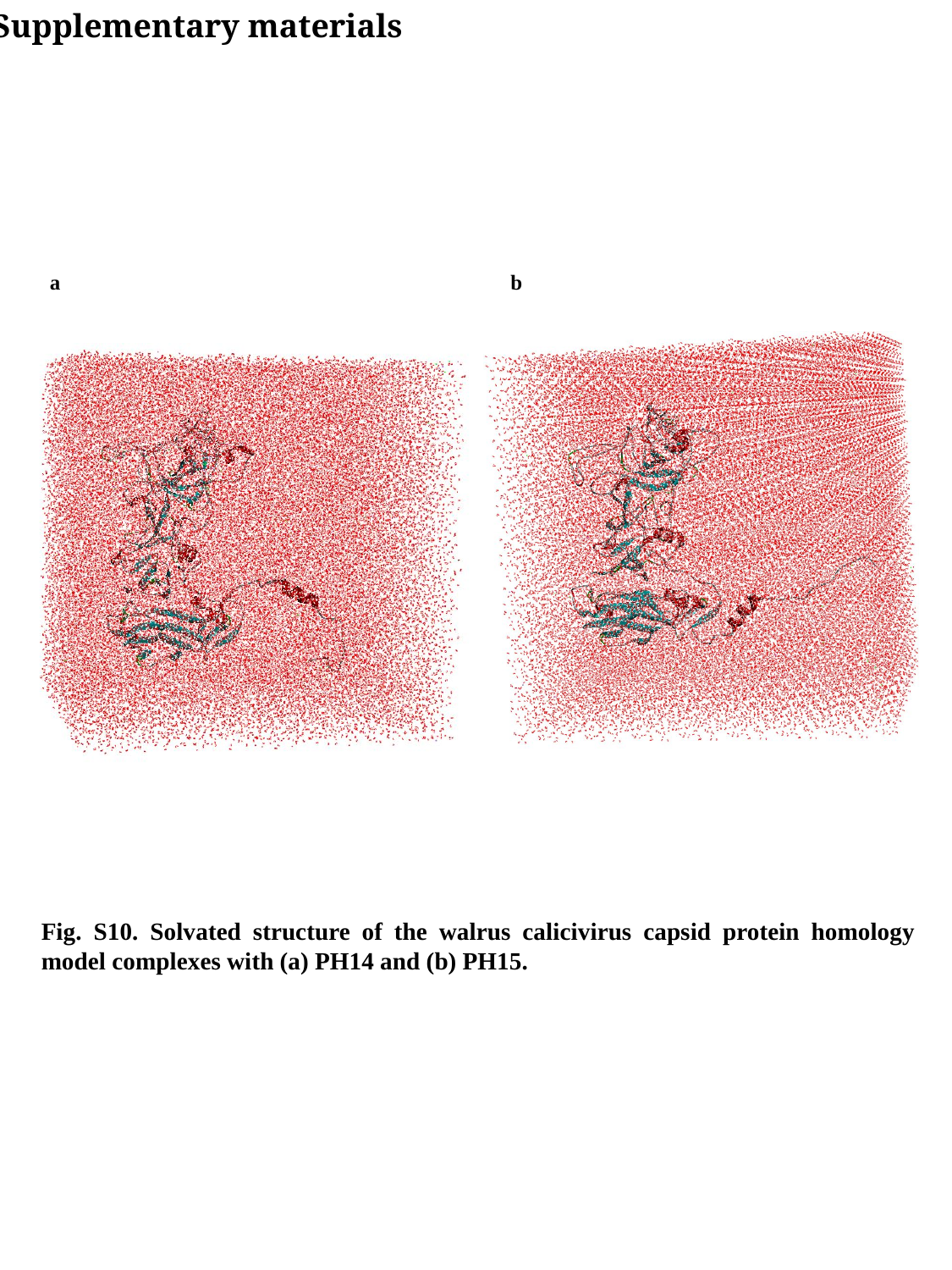

Supplementary materials
a
b
Fig. S10. Solvated structure of the walrus calicivirus capsid protein homology model complexes with (a) PH14 and (b) PH15.

## Slide 11
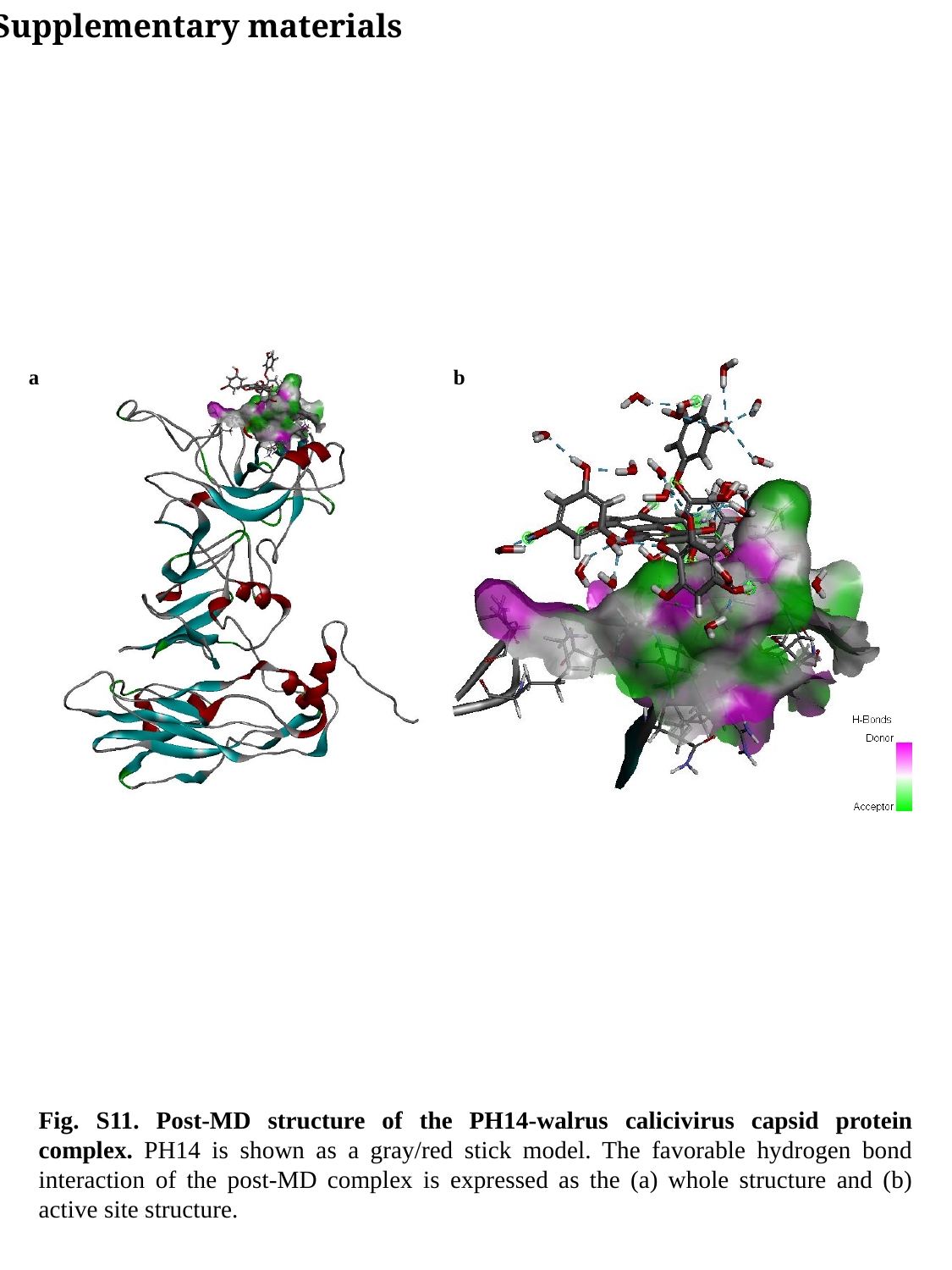

Supplementary materials
a
b
Fig. S11. Post-MD structure of the PH14-walrus calicivirus capsid protein complex. PH14 is shown as a gray/red stick model. The favorable hydrogen bond interaction of the post-MD complex is expressed as the (a) whole structure and (b) active site structure.

## Slide 12
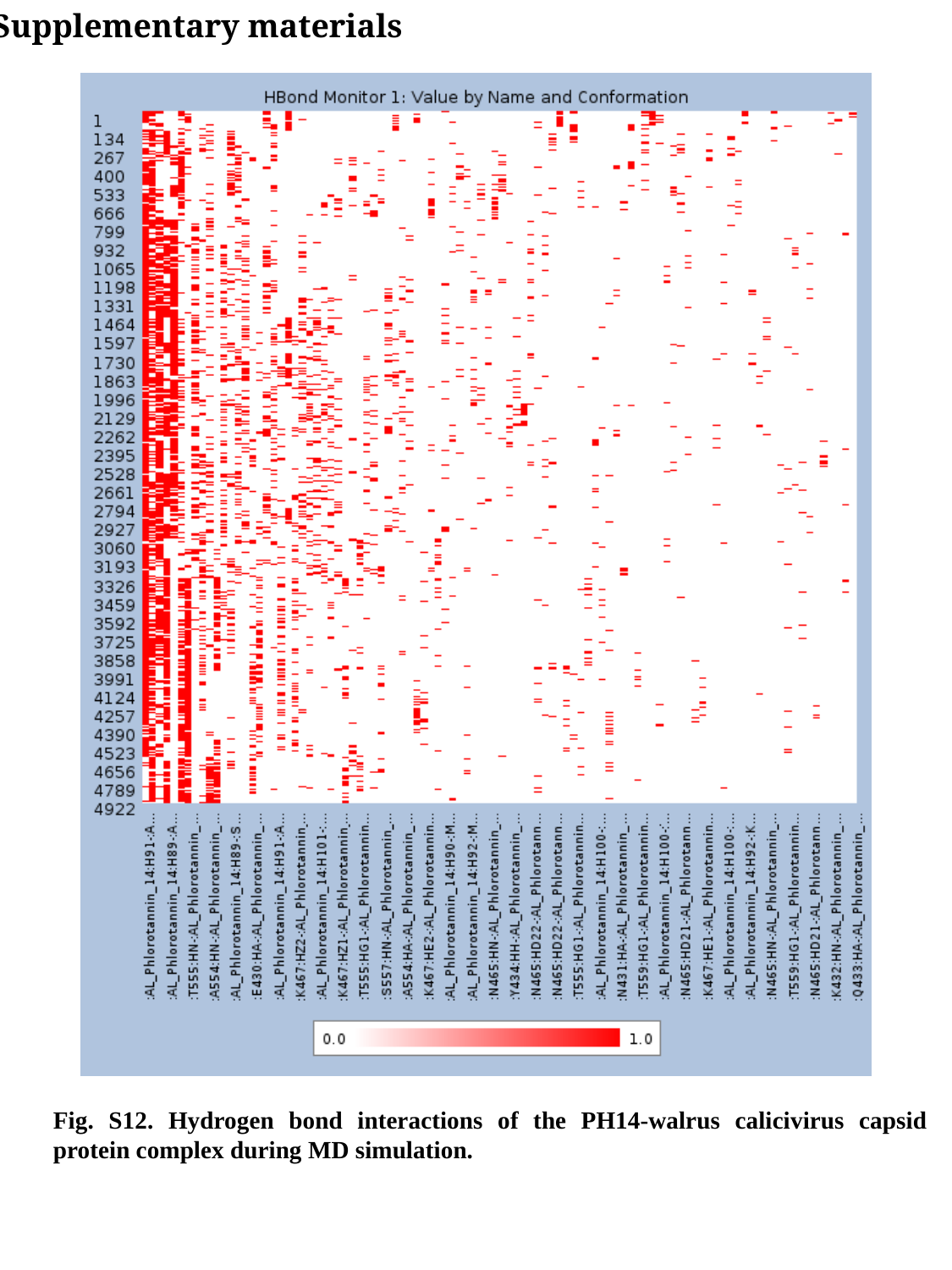

Supplementary materials
Fig. S12. Hydrogen bond interactions of the PH14-walrus calicivirus capsid protein complex during MD simulation.
