# Supplementary material for: Structure-Based In Silico Screening of Marine Phlorotannins for Potential Walrus Calicivirus Inhibitor
Source: Int J Mol Sci. 2023 Oct 30;24(21):15774. doi: 10.3390/ijms242115774 (PMC10647355; doi:10.3390/ijms242115774)
Supplement: Supplementary file 1 [file ijms-24-15774-s001.zip › supplementary tables S1-S6.pdf]

Table S1. PDF total energy, PDF physical energy, and DOPE scores of the 20 generated models of walrus calicivirus capsid protein

Model	PDF total energy	PDF physical energy	DOPE score
NP_777371_1.M0003	20760.0176	1699.0670	-53546.2969
NP_777371_1.M0020	20831.1992	1718.0660	-53855.3359
NP_777371_1.M0018	20875.7539	1724.0704	-53901.8359
NP_777371_1.M0017	20897.7461	1719.4126	-53345.3242
NP_777371_1.M0016	20914.7461	1712.2336	-53445.5234
NP_777371_1.M0008	20950.1289	1721.3962	-53625.5703
NP_777371_1.M0010	21004.2676	1732.9737	-53558.2930
NP_777371_1.M0019	21038.2910	1753.0573	-53661.4492
NP_777371_1.M0014	21099.1582	1698.7029	-53792.7422
NP_777371_1.M0004	21113.0723	1737.2835	-53723.1719
NP_777371_1.M0006	21122.4707	1713.6365	-53821.2813
NP_777371_1.M0015	21178.4902	1766.1433	-53534.6094
NP_777371_1.M0001	21193.1875	1761.6280	-53544.9727
NP_777371_1.M0007	21344.6855	1821.2846	-53810.6094
NP_777371_1.M0012	21484.3340	1734.4843	-53407.6563
NP_777371_1.M0011	21901.2285	1793.4429	-52370.1563
NP_777371_1.M0005	21903.2793	1792.9699	-52611.4141
NP_777371_1.M0013	22006.4746	1807.4530	-53013.9453
NP_777371_1.M0009	22139.9883	1780.3487	-52428.6406
NP_777371_1.M0002	22374.4180	1843.5933	-52471.3828

PDF, probability density function; DOPE, discrete optimized protein energy

Table S2. Number of interaction energy of complexes of phlorotannins with the walrus calicivirus capsid protein homology model

PHs	Hydrogen bond			Electrostatic			Hydrophobic				Other
	CV HB[1)]	CB HB[2)]	PD HB[3)]	PC[4)]	PA[5)]	PS[6)]	AP[7)]	PA[8)]	PPT[9)]	PPS[10)]	PL[11)]
PH1	8	2		1			1	1			
PH2	4	1	1								
PH3	2						1		1		
PH4	1										
PH5	9		2						1		
PH6	6			1				2			
PH7	5							1	1	1	
PH8	9	3			1				1		
PH9	3										
PH10	4	2					1	1			
PH11	9	2						1			1
PH12	5	3									
PH13	6	3						2	1		
PH14	8	1	1					5			1
PH15	10	2	1	1	1			7	1		
PH16	6	2						1	1	1	1
PH17	1							1			

[1)]Conventional hydrogen bond, [2)]Carbon–hydrogen bond, [3)]Pi-Donor hydrogen bond, [4)]Pi-Cation, [5)]Pi-Anion, [6)]Pi-Sigma, [7)]Amide-Pi Stacked, [8)]Pi-Alkyl, [9)]Pi-Pi T-shaped, [10)]Pi-Pi Stacked, [11)]Pi-Lone Pair

Table S3. Interaction energy (kcal/mol) of complexes of phlorotannins with the walrus calicivirus capsid protein homology model

PHs	Binding Energy	Ligand Energy	Complex Entropy	Protein Entropy	Ligand Entropy	-CDOCK Energy	-CDOCK Interaction Energy
PH1	-235.579	53.282	-32.7004	-32.6865	-19.9543	28.9498	48.0255
PH2	-98.642	20.407	-32.6902	-32.6857	-16.9862	20.3369	24.4961
PH3	-152.331	100.694	-32.6942	-32.6833	-19.9496	13.4974	32.7946
PH4	-73.108	59.430	-32.6872	-32.6765	-19.9600	15.8748	24.3909
PH5	-248.713	80.349	-32.7052	-32.6864	-20.7317	30.1543	52.9435
PH6	-229.466	62.967	-32.7041	-32.6870	-20.8116	7.84854	27.4738
PH7	-290.683	74.643	-32.6990	-32.6843	-20.6516	10.7334	35.3716
PH8	-368.514	91.408	-32.7222	-32.6928	-22.0084	21.9745	51.5065
PH9	-195.824	-70.518	-32.7231	-32.6898	-21.8351	18.3312	30.0503
PH10	-274.292	53.334	-32.7113	-32.6873	-21.2858	11.2519	41.7174
PH11	-198.765	65.271	-32.7081	-32.6836	-21.3211	12.0480	41.7175
PH12	-345.507	76.922	-32.7173	-32.6873	-21.8525	25.4066	58.9415
PH13	-292.850	86.658	-32.7150	-32.6828	-21.8557	13.0352	44.4525
PH14	-405.571	125.346	-32.7327	-32.6922	-22.5270	3.4846	58.4701
PH15	-406.115	101.838	-32.7273	-32.6860	-22.5623	4.8323	52.5371
PH16	-368.962	114.701	-32.7179	-32.6914	-22.2586	1.2320	40.1138
PH17	-266.837	66.300	-32.6997	-32.6855	-20.8861	11.0758	34.2722

CDOCKER: CHARMM-based DOCKER

Table S4. List of binding interactions of PH14 with the walrus calicivirus capsid protein homology model

Interactions	Distance	Types
N465:HD22 - PH14:O36	2.6757	Conventional Hydrogen Bond
N465:HD22 - PH14:O65	1.9193	Conventional Hydrogen Bond
R553:HH21 - PH14:O59	1.9975	Conventional Hydrogen Bond
PH14:H84 - T555:OG1	2.4982	Conventional Hydrogen Bond
PH14:H89 - K432:O	2.7280	Conventional Hydrogen Bond
PH14:H90 - S557:O	2.4969	Conventional Hydrogen Bond
PH14:H91 - A554:O	1.8299	Conventional Hydrogen Bond
PH14:H92 - N431:OD1	1.7948	Conventional Hydrogen Bond
S429:HB1 - PH14:O58	2.3495	Carbon–Hydrogen Bond
PH14:H99 - PH14	2.8356	Pi-Donor Hydrogen Bond
A554:O - PH14	2.7819	Pi-Lone Pair
PH14 - L435	5.0111	Pi-Alkyl
PH14 - R553	5.3391	Pi-Alkyl
PH14 - M556	5.2507	Pi-Alkyl
PH14 - A554	5.1978	Pi-Alkyl
PH14 - A554	4.6897	Pi-Alkyl

Table S5. List of binding interactions of PH15 with the walrus calicivirus capsid protein homology model

Interactions	Distance	Types
R553:HH12 - PH15:O70	2.3859	Conventional Hydrogen Bond
PH15:H88 - PH15:O65	1.8906	Conventional Hydrogen Bond
PH15:H90 - T555:OG1	1.8787	Conventional Hydrogen Bond
PH15:H93 - D551:OD2	2.0815	Conventional Hydrogen Bond
PH15:H93 - D551:O	2.7941	Conventional Hydrogen Bond
PH15:H99 - R553:O	2.2082	Conventional Hydrogen Bond
PH15:H99 - A554:O	2.2155	Conventional Hydrogen Bond
PH15:H100 - K558:O	1.7855	Conventional Hydrogen Bond
PH15:H101 - K432:O	1.9641	Conventional Hydrogen Bond
PH15:H101 - Y434:O	2.2840	Conventional Hydrogen Bond
M556:HA - PH15:O50	2.7485	Carbon–Hydrogen Bond
M556:HA - PH15:O69	2.5581	Carbon–Hydrogen Bond
R553:NH1 - PH15	3.9104	Pi-Cation
D512:OD2 - PH15	4.3313	Pi-Anion
PH15:H98 - PH15	2.6574	Pi-Donor Hydrogen Bond
PH15 - PH15	5.7702	Pi-Pi T-shaped
PH15 - A554	5.0571	Pi-Alkyl
PH15 - A554	4.1989	Pi-Alkyl
PH15 - A554	4.5137	Pi-Alkyl
PH15 - A554	3.9187	Pi-Alkyl
PH15 - A554	5.1485	Pi-Alkyl
PH15 - M556	4.5782	Pi-Alkyl

Table S6. Comparison of the interaction energies of feline junctional adhesion molecule A–capsid protein complexes

Amino acids	Interaction energy (kcal/mol)		
	Ligand-free	PH14-	PH15-
G_SER33	-5.432641	-23.943008	-19.565445
G_GLU34		-13.494718	-46.029301
G_PRO35		-7.525343	
G_ASP36		-22.548445	
G_VAL37		-6.847295	
G_ARG38		-13.021039	-34.698479
G_VAL39			-8.376345
G_LYS46		-0.616595	-29.269098
G_SER50	8.546897		
G_LEU128		0.19758	-0.675931
G_TYR217			-14.910054
H_TYR31	-25.236763		
H_SER33	-8.927774		-32.423805
H_GLU34		-24.442978	-62.721897
H_ASP36		-31.803093	
H_VAL37		0.601529	
H_ARG38		-19.49382	-18.157997
H_PRO40	12.587757		
H_ASP42	48.146461	-106.17218	-104.632706
H_LYS43	-51.85257	1.598235	17.608675
H_PRO44	-13.527664	-32.494175	-18.950596
H_ALA45	-13.208787	-2.968642	-16.14814
H_LYS46	-61.305534	-158.143906	-132.702286
H_SER48		-31.674452	

Amino acids	Interaction energy (kcal/mol)		
	Ligand-free	PH14-	PH15-
H_SER50	-4.56848	-15.169076	
H_TYR51	-7.869105	-2.243268	-15.295883
H_SER52	0.311664		-20.479977
H_GLY53	-9.715839		
H_PHE54	-13.689794		
H_SER55	8.429708	-6.568112	-7.548132
H_ASN56		-10.811067	-4.177859
H_PRO57	5.917749		-4.315956
H_THR87		-14.985053	
H_SER89	-12.203172	-16.159328	-16.343271
H_HIS90	-8.224008	-7.834162	-5.136015
H_SER91	5.27427	-17.307737	-20.457642
H_GLY92			-12.710009
H_THR94	-5.823633	-15.982031	-23.379801
H_PHE95		4.082521	-6.025679
H_HIS96	8.296247	1.128721	-10.854245
H_SER97	8.218183		
H_PRO130	7.408176		
H_TYR217	-0.429157		
H_GLY218	-3.257852		
H_MET219	-4.377364		
Total	-136.51303	-594.64093	-668.37787
